# DuSAFNet: A Multi-Path Feature Fusion and Spectral–Temporal Attention-Based Model for Bird Audio Classification

**DOI:** 10.3390/ani15152228

**Published:** 2025-07-29

**Authors:** Zhengyang Lu, Huan Li, Min Liu, Yibin Lin, Yao Qin, Xuanyu Wu, Nanbo Xu, Haibo Pu

**Affiliations:** 1 College of Information Engineering, Sichuan Agriculture University, Ya’an 625014, China; 202205931@stu.sicau.edu.cn (Z.L.); chihuan@stu.sicau.edu.cn (H.L.); 202308511@stu.sicau.edu.cn (M.L.); 202005861@stu.sicau.edu.cn (Y.Q.); jonyzepplie@stu.sicau.edu.cn (N.X.); 2 College of Fisheries, Jimei University, Xiamen 361021, China; 202221062084@jmu.edu.cn; 3 College of Computer and Control Engineering, Northeast Forestry University, Harbin 150040, China; 2022220280@nefu.edu.cn; 4 Ya’an Key Laboratory of Intelligent Prevention and Control of Crop Diseases and Pests, Ya’an 625000, China

**Keywords:** bird audio classification, spectral–temporal attention, multi-path feature fusion, ArcMarginProduct, passive acoustic monitoring, real-time conservation

## Abstract

This research presents DuSAFNet, a lightweight deep neural network for fine-grained bird audio classification. DuSAFNet combines dual-path feature fusion, spectral–temporal attention, and a multi-band ArcMarginProduct classifier to enhance inter-class separability and capture both local and global spectro–temporal cues. Unlike single-feature approaches, DuSAFNet captures both local spectral textures and long-range temporal dependencies in Mel-spectrogram inputs and explicitly enhances inter-class separability across low, mid, and high frequency bands. On a curated dataset of 17,653 three-second recordings spanning 18 species, DuSAFNet achieves 96.88% accuracy and a 96.83% F1 score using only 6.77 M parameters and 2.275 GFLOPs. Cross-dataset evaluation on Birdsdata yields 93.74% accuracy, demonstrating robust generalization to new recording conditions. Its lightweight design and high performance make DuSAFNet well-suited for edge-device deployment and real-time alerts for rare or threatened species. This work lays the foundation for scalable, automated acoustic monitoring to inform biodiversity assessments and conservation planning.

## Simple Summary

Bird populations serve as vital indicators of ecosystem health, yet traditional survey methods—whether listening by ear or using simple automated detectors—are laborious, error-prone, and struggle in noisy environments. In this work, this research introduces DuSAFNet, a deep learning model that leverages multi-path feature fusion and spectral–temporal attention to automatically recognize 18 common bird species from three-second audio clips with over 96% accuracy. By overcoming limitations of single-feature approaches and operating robustly under real-world noise, our method enables rapid, large-scale acoustic monitoring to support population assessments, habitat management, and conservation planning.

## 1. Introduction

Biodiversity is a critical component for maintaining the stability and adaptability of ecosystems, and its effective monitoring is particularly crucial in the context of global climate change and the intensification of human activities [1]. As one of the most environmentally sensitive biological groups within ecosystems, birds are widely regarded as ecological indicator species and play a pivotal role in ecosystem health assessments and biodiversity conservation efforts [2]. Birds exhibit rapid responses to factors such as habitat quality, land-use changes, and climate fluctuations, making their population dynamics valuable indicators of ecosystem trends. In recent years, bird populations and biodiversity worldwide have sharply declined due to the combined effects of human activities and environmental changes [3]. Consequently, enhancing bird monitoring capabilities, strengthening conservation mechanisms, and safeguarding the healthy development of bird populations have become increasingly vital.

Birds serve not only as biodiversity indicators but also as sentinels of habitat integrity, since many species exhibit distinct seasonal and behavioral vocal patterns [4,5]. For example, migratory waterfowl such as Canada Goose produce conspicuous honking sequences during spring courtship and territorial displays [6], whereas Whooper Swan deploys low-frequency contact calls during molting periods. Such phenological shifts in acoustic signals reflect underlying physiological and ecological processes—breeding readiness, flock cohesion, or predation risk [7]—and offer a non-invasive window into population health and ecosystem function.

Birds typically possess ecological traits such as species richness, prominent vocalizations, and high diurnal activity, which render them highly observable and representative in field surveys and long-term ecological monitoring [8]. Notably, the species information, behavioral patterns, and temporal rhythms embedded in bird vocalizations provide a crucial data source for eco-acoustic research [9]. Through continuous monitoring of avian soundscapes, researchers can obtain essential ecological data without disturbing natural behaviors, thereby offering a more comprehensive assessment of ecosystem structure and functionality [10].

Traditional bird monitoring methods primarily include visual observation techniques [2] and manual acoustic recording methods [9]. These methods rely on human experts for species identification and the interpretation of bird calls [8]. However, manual and visual surveys suffer from several drawbacks. They are time-consuming and labor-intensive, hindering large-scale, long-term studies. Observer expertise varies, introducing subjective bias. Surveys may also disturb bird behavior. Finally, many species call primarily at dawn, dusk, or in concealed habitats—further complicating manual monitoring [10].

With the development of digital recording equipment and sensor technologies, passive acoustic monitoring (PAM) has rapidly become an effective tool in wildlife research [11]. This method involves deploying automatic recorders in the field to continuously collect natural environmental sounds, enabling continuous monitoring of bird soundscapes [12].

Despite the promise of passive acoustic monitoring (PAM), several ecological challenges persist in field deployment. Ambient soundscapes vary dramatically with habitat type—open wetlands, forest understories, and agricultural mosaics each impose distinct noise profiles (water flow, wind rustle, machinery hum) that can mask target vocalizations. Moreover, many species concentrate their calling activity at dawn and dusk or in concealed microhabitats, further complicating manual surveys and naïve-automated detectors. Therefore, robust signal processing and intelligent classification methods are essential to disentangle overlapping sources, adapt to variable soundscapes, and ultimately translate raw acoustic data into actionable ecological insights [13].

Before the rise of deep learning, bird audio recognition primarily relied on manually extracted features combined with traditional machine learning models. Typical approaches included template matching methods (e.g., Dynamic Time Warping (DTW)) [14] and feature-based classification methods such as Gaussian Mixture Models (GMM) [15], Hidden Markov Models (HMM) [16], Support Vector Machines (SVM) [17], and Random Forests (RF) [18]. These methods reduced human subjectivity to some extent, but they often struggled to achieve high accuracy when dealing with the highly variable and complex bird calls in field recordings [19]. Traditional methods, which rely on manually selected acoustic features such as Mel-frequency cepstral coefficients (MFCC) and Linear Predictive Cepstral Coefficients (LPCC), face difficulties in capturing the complex patterns and long-range dependencies in bird calls, thus limiting the performance and generalization ability of the models [20]. With the increase in data scale and environmental noise, the limitations of these traditional approaches have become more pronounced, highlighting the urgent need for more advanced methods to improve recognition robustness and accuracy.

In recent years, owing to advancements in deep learning technologies, neural network-based bird audio recognition systems have emerged prolifically. The introduction of convolutional neural networks (CNNs) has significantly enhanced the performance of bird audio recognition. Researchers typically treat the time–frequency spectrum of bird calls (e.g., spectrograms or spectrographs) as image inputs, with CNNs automatically extracting high-recognition time–frequency features [21]. Compared with handcrafted features, CNNs can learn local patterns in the bird call frequency spectrum (such as frequency modulation and harmonic structures) through convolutional filters, effectively capturing the temporal and frequency local structures of bird calls [21]. In early applications, shallow CNNs demonstrated the potential to surpass traditional methods. For example, in the BirdCLEF2016 challenge, models that used convolutional networks to process spectrograms set a new record of 55%, validating the superiority of deep learning methods over traditional approaches [22]. With the deepening of networks and the development of data augmentation techniques, more complex CNN architectures have achieved superior performance: Sankupellay et al. employed the ResNet-50 network to identify 46 bird species, achieving an accuracy of 72%, significantly higher than earlier traditional methods [23]. A study using the Inception-v3 network on the BirdCLEF2019 dataset, which encompasses 695 bird species, resulted in a final accuracy of only approximately 16% [24]. But this also revealed a limitation of CNNs: Due to the invariance of convolution to frequency translation, CNNs struggle to distinguish similarly shaped calls when they occur at different frequency bands [11]. This phenomenon highlights a gap in modeling along the frequency axis—traditional CNNs are more focused on local shape patterns and lack sensitivity to absolute frequency positions [11]. Overall, CNN-based deep learning methods have significantly outperformed earlier techniques in bird audio recognition, although their local perceptual characteristics present new challenges.

Recently, several studies have explored wavelet-based time–frequency representations in bioacoustic analysis. Andén and Mallat [25] introduced the wavelet scattering transform with Morlet wavelets to extract stable, deformation-invariant features from non-stationary signals, achieving state-of-the-art performance in limited-label audio classification. More recently, Gauthier et al. [26] proposed parametric scattering networks that learn wavelet filter parameters in a data-driven fashion, demonstrating improved species classification accuracy under varying noise conditions. These findings suggest that wavelet-based methods can capture rapid frequency modulations and non-stationary chirp patterns, complementing STFT-derived spectrograms for bird audio classification.

Bird calls exhibit distinct temporal properties, with variations in rhythm, syllable order, and duration across different species. Recurrent neural networks (RNNs) and their variants (long short-term memory (LSTM) networks and gated recurrent units (GRUs)) are adept at modeling sequential data, capturing temporal dependencies in bird call audio [27]. Unlike CNNs, which focus solely on instantaneous spectral features, RNNs can memorize and utilize the state changes in bird calls over time, such as the sequence and duration of notes, thereby enhancing classification performance for complex calls [21]. To simultaneously address spectral local feature extraction and temporal modeling, the convolutional–recurrent hybrid model (CRNN) was introduced [28]. CRNNs typically use CNNs as the frontend to extract spectral features from each short-time frame, followed by RNNs to model these feature sequences, exhibiting superior performance in bird sound recognition. Gupta et al. compared various convolutional–recurrent networks on large bird call datasets, finding that hybrid models incorporating LSTM/GRU performed the best on large-scale data such as the Cornell Bird Call Challenge [21]. Some studies have also introduced attention mechanisms to enhance the RNN’s focus on critical information: Noumida et al. constructed a hierarchical attention BiGRU model, achieving favorable results in multi-label bird species recognition. The attention mechanism enabled the model to focus on the most relevant time segments in the audio, thus improving classification accuracy [29]. In summary, RNN and CRNN methods have addressed the shortcomings of pure CNNs in temporal sequence modeling, achieving remarkable progress in the sequential modeling of bird audio. By combining convolutional and recurrent networks, the model can more comprehensively represent bird call signals, achieving higher recognition performance than single-architecture approaches.

Over the past few years, with the remarkable success of transformer models in natural language processing and computer vision, their application has gradually extended to bird audio recognition. The transformer architecture, relying on its self-attention mechanism, enables the global modeling of dependencies across any positions in a sequence, which is particularly advantageous for capturing long-term correlated patterns across both time and frequency in bird calls [30]. Early attempts include Puget’s STFT-Transformer model [31], which used logarithmic Mel-spectra as input for the transformer, achieving impressive results in BirdCLEF2021. The model outperformed traditional CNN baselines in both recognition accuracy and processing speed. Furthermore, Tang et al. developed the Transound model, incorporating a Vision Transformer with an exceptionally large number of attention heads to encode bird call features such as Mel-frequency cepstral coefficients (MFCC). This model demonstrated a 10.64% increase in accuracy compared with the best existing CNN model [32], showcasing the significant advantages of the transformer in bird call recognition tasks and its ability to uncover discriminative patterns that CNNs fail to capture. Additionally, some studies have combined transformer with convolutional networks to form hybrid architectures. For instance, Xiao et al. integrated multiple features—such as MFCC, chroma spectrograms, and spectral centroid—into an enhanced ResNet (AM-ResNet) and transformer module, achieving a recognition accuracy of 90.1% [33]. Zhang et al. merged features extracted by deep CNNs from logarithmic Mel-spectra with features encoded by transformers (MFCC/chroma) to achieve an accuracy of 97.99% in classifying 20 bird species [19]. These studies indicate that the transformer not only serves as an independent time–frequency modeler but, when combined with traditional CNNs, further enhances performance. The introduction of transformer-based methods has endowed bird audio recognition with the capability of global modeling, effectively mitigating the limitations of CNNs’ local receptive fields. This has proven especially beneficial for distinguishing cross-time and cross-frequency related patterns in long recordings or complex soundscapes.

Given the complex and dynamic patterns in bird calls, models based on a single architecture often fail to comprehensively capture all discriminative information. Consequently, various hybrid architectures and feature fusion strategies have emerged in recent years to combine the advantages of different models and features. For example, Liu et al. proposed a multi-scale convolutional model (EMSCNN), which uses convolutional kernels of varying sizes to extract both detailed and overall features of bird calls, then concatenates multi-scale features, achieving a recognition accuracy of 91.49% for 30 bird species [34]. However, this model has a large number of parameters and simply concatenates the outputs of different convolution branches, lacking a mechanism for further exploring the correlations between features at different scales. The integration of attention mechanisms into deep learning models can guide the model’s focus on more important feature dimensions or time–frequency locations, which has also been increasingly applied in bird audio recognition. For instance, Gunawan et al. integrated the Convolutional Block Attention Module (CBAM) into convolutional networks for owl call classification, achieving superior performance compared with CNNs without attention [35]. Another study designed the “MFF-ScSEnet” network, which combines multi-scale feature fusion with scSE attention, reaching over 96% accuracy in bird call classification [36]. However, most of the currently applied attention modules focus on weighting the channel or spatial (time–frequency) dimensions, with few efforts focusing on separately designing attention mechanisms for the time and frequency axes. Existing methods treat the entire time–frequency plane as a spatial dimension, which may fail to distinguish which frequencies at which times are more important.

To address the critical limitations of prior methods—namely, the insufficient joint modeling of local and global features, the lack of integrated spectral–temporal attention mechanisms, and the suboptimal discriminative capacity due to simplistic feature fusion—this paper proposes DuSAFNet (Dual-path spectro–temporal Attention & Fusion Network) for bird sound recognition. The proposed **DPFM** is built upon a shared backbone, employing a dual-path dense-residual skip structure to facilitate multi-scale feature extraction. This architecture is complemented by a **Spectral–Temporal Attention (STA)** mechanism, which allows the network to selectively focus on key frequency bands and temporal windows, capturing both local and global features. To further enhance feature integration, **Gated Fusion Mapping (GFM)** is utilized, enabling dynamic and context-sensitive fusion of features across the two paths. Additionally, the **Temporal-Spatial Multi-scale Attention Module (TSFM)** provides deep contextual modeling of the features, enriching their temporal and spatial relationships. Finally, the integration of a multi-band **ArcMarginProduct** classifier further refines the model’s ability to distinguish between complex audio patterns, improving recognition accuracy across various bird species. The key innovations and contributions of this work are summarized as follows:

(i) **Dual-Path Feature Module (DPFM)**
  A dual-path feature extraction module, comprising **GrowthBranch** and **SkipBranch**, is designed to extract features in parallel. The former employs dense growth units to capture fine-grained local textures, while the latter uses a residual skip structure to capture long-range context. This dual-path approach facilitates complementary modeling of features at different scales, enabling collaborative perception of time–frequency features in bird song.
(ii) **Spectral–Temporal Attention (STA)**
  Attention weights are modeled separately on the frequency axis and time axis, allowing the network to automatically focus on the most discriminative frequency bands and time periods during training. This effectively addresses the limitations of traditional convolutional networks, which fail to adequately capture absolute frequency information and long-range temporal relationships.
(iii) **Gated Fusion Map (GFM)**
  A lightweight gating mechanism is proposed, which dynamically adjusts the proportion of information flow from each branch during the fusion process. This self-adaptive approach suppresses redundant features while highlighting critical information, thereby enhancing the fusion efficiency and feature quality.
(iv) **Temporal-Spatial Fusion Module (TSFM)**
  The **LocalSpanAttention** (local span temporal attention) and **MultiscaleAttentionModule** (multi-scale spatial-channel attention) are coupled to achieve unified modeling of local dependencies in the time domain and multi-scale re-scaling in the spatial-channel domain. This comprehensive approach enhances the recognition capability of the bird song spectrogram along both temporal and spatial dimensions.
(v) **Multi-band ArcMarginProduct Classifier**
  The feature maps are divided into low, medium, and high frequency bands based on their height. The model leverages species-specific differences in bird calls across these frequency bands by using **ArcMarginProduct** with different scale factors and angular margins for classification. These features are weighted and fused using learnable weights, which explicitly increase the inter-class angular distance and improve fine-grained species discrimination.

## 2. Materials and Methods

### 2.1. Dataset

The avian audio data used in this study were sourced from the internationally recognized, open-access bird song database, Xeno-Canto (https://www.xeno-canto.org). All audio data used in this research are publicly available from the Xeno-Canto database (https://xeno-canto.org/). Recordings were collected by volunteers in the field using non-invasive microphones. No live animals were captured, processed, or experimentally manipulated, and no human subjects or personally identifiable information were involved. To assess potential site and device biases, the selected Xeno-Canto recordings span 15 countries across five biogeographic regions, recorded with diverse equipment. Device metadata were logged, and all audio files were standardized to mono 16 kHz to mitigate sampling-rate and microphone-response variations. Initially, search parameters were set to select recordings with a quality rating above “C” to ensure high audio quality. The retrieved audio files were then subjected to a metadata review covering recording duration, environmental conditions, and geographical coordinates, ensuring that the data captured diverse real-world background noise and were well distributed across various sampling regions. As a result, a total of 2128 raw audio files were obtained, encompassing 9 orders, 11 families, 17 genera, and 18 species. The duration of these raw recordings varied, ranging from a few seconds to several minutes.

To enhance the accuracy of subsequent analysis and ensure the stability of model training, this study adopted widely used audio preprocessing methods from existing literature. Additionally, custom preprocessing techniques were developed for the bird audio features collected in this study. The preprocessing pipeline was designed in several stages. Initially, all raw recordings were converted to mono-channel WAV format and resampled to 16 kHz [1], meeting the sampling rate requirements for standard bird song research and deep learning model inputs. Following this, based on the experience of Kahl et al. [37], who annotated 80,000 bird calls, a two-threshold Root Mean Square (RMS) energy method was used for Voice Activity Detection (VAD) [27]. The high and low thresholds were set at 0.002 and 0.0005, respectively. This method accurately identified high-energy frames containing bird call signals, extending the frame intervals forward and backward to the lower threshold region, which preserved short, sharp calls while effectively removing long silent periods and background noise. Finally, the VAD-processed audio was segmented into non-overlapping 3 s windows [20]. Segments shorter than 3 s or lacking distinct bird calls were discarded. Through this process, the original 2128 multi-segment recordings were transformed into 17,653 uniformly sized 3 s audio clips.

To guarantee that our dataset contains only single-source bird vocalizations, we filtered Xeno Canto metadata for single-species recordings and manually inspected each spectrogram to exclude any clips with overlapping calls. While we retained subtle ambient background noise to improve DuSAFNet’s robustness in real-world conditions, recordings exhibiting severe environmental interference or multiple species were removed. Consequently, the final corpus comprises 17,653 clean, single-source 3 s bird audio segments.

To avoid training imbalances due to an overrepresentation of species with a high number of recordings, audio clips for each species were sorted by filename order to preserve recording chronology, retaining only the first 1000 clips per species (species with fewer than 1000 clips were retained in their entirety). After this balancing step, a total of 17,653 audio clips were retained. For each of the preserved 3 s segments, Short-Time Fourier Transform (STFT) [1] was applied using a 25 ms Hanning window with a 10 ms frame shift. The time–frequency spectrogram was then mapped to the Mel frequency domain using a 128-channel Mel filter bank [37] and log-transformed to compress the dynamic range and enhance detail contrast [27]. The resulting log Mel spectrograms were rendered into 224 × 224 pixel, three-channel color images, organized in directories by species.

Next, to ensure fairness in model evaluation and reproducibility of experimental results, a random splitting method was employed [20]. The audio clips were randomly divided into training (12,357 clips) and testing (5296 clips) sets with a 70:30 ratio.

The constructed avian audio dataset includes a diverse range of bird species and a wide variety of acoustic environments. After thorough standardization and balancing, the dataset is well-equipped to support subsequent bird song classification and recognition tasks. The specific composition of the dataset is shown in Table 1.

### 2.2. Data Preprocessing

To enable DuSAFNet to accurately recognize bird calls in complex field soundscapes, this study has designed a rigorous, end-to-end preprocessing pipeline with well-defined mathematical formulations. The pipeline encompasses four key stages: audio normalization and segmentation, Mel spectrogram generation, spectrogram-level enhancement, and image-level data augmentation. This process standardizes the input format across different recording devices and environments while enhancing the model’s robustness to noise, signal loss, and device variations through multiple augmentations.

#### 2.2.1. Audio Normalization and Effective Segment Segmentation

To mitigate resolution discrepancies introduced by varying recording devices and environments, and to eliminate prolonged silent intervals and irrelevant noise, we performed uniform normalization and segmentation on each raw audio signal x[n]. For the original bird recordings x[n] collected from Xeno-Canto, we first employed Librosa to load and resample the signals to a 16 kHz mono format, ensuring consistency in both time-domain and frequency-domain resolution. Subsequently, using a window length of 400 samples (approximately 25 ms) and a frame shift of 160 samples (approximately 10 ms), we calculated the Root Mean Square (RMS) energy for each frame:

(1) $$E\left(n\right)=\sqrt{\frac{1}{L}\sum_{i=0}^{L-1}x{\left(n+i\right)}^{2}}$$

where E(n) represents the energy of the *n*-th frame, x[n] is the resampled mono signal sample value, and *L* is the analysis window length.

This research then applied a two-threshold Voice Activity Detection (VAD) technique, setting high and low energy thresholds at $T_{h}=0.002$ and $T_{l}=0.0005$, respectively. Frames with $E\left(n\right)>T_{h}$ were labeled as “high-energy bird call frames,” and each high-energy frame triggered an extension forward and backward to neighboring frames where $E\left(n\right)>T_{l}$, generating continuous bird call segments. This approach effectively preserved transient vocalizations while suppressing silence and background noise. Each continuous bird call segment selected by VAD was then segmented into non-overlapping 3 s windows:

(2) $$segment_{k}=x\left[kT:\left(k+1\right)T\right],\;T=3\;s,\;k=0,1,\ldots$$

where $segment_{k}$ denotes the *k*-th window segment, and *T* is the window length (in seconds). Only segments that were exactly 3 s in length were retained, and segments shorter than 3 s or lacking any high-energy frames were discarded. This process resulted in the normalization of the original recordings into a series of balanced, denoised, fixed-length bird call segments, providing high-quality and standardized inputs for subsequent Mel spectrogram generation and deep network training.

#### 2.2.2. Mel Spectrogram Generation and Logarithmic Transformation

After audio segmentation, each 3 s bird call segment undergoes Short-Time Fourier Transform (STFT) to reveal the time–frequency structure of the signal. Specifically, with a window length L=400 samples (25 ms), a frame shift Δ=160 samples (10 ms), and N=512 FFT points, the complex spectrum of the *t*-th frame at frequency *f* is computed as

(3) $$X\left(f,t\right)=\sum_{m=0}^{L-1}x\left(t+m\right)w\left(m\right)e^{-j2\pi fm/N}$$

The selection of a 25 ms analysis window (400 samples) and a 10 ms frame shift (160 samples) balances time–frequency resolution for bird vocalizations. Although birds lack phonemic segmentation, our experiments demonstrate that 20–30 ms windows sufficiently resolve harmonic and formant-like structures in bird calls. A 10 ms shift yields ∼60% overlap, ensuring smooth tracking of rapid frequency modulations without excessive redundancy or computational overhead. Tests with shorter windows (10–15 ms) or larger overlaps (≥75%) offered no appreciable accuracy gains but increased processing cost. Thus, our parameters achieve an optimal trade-off among spectral detail, temporal continuity, and efficiency.

Where x[n] represents the resampled 16 kHz mono signal, w(m) is the Hanning window function, and *N* is the FFT point number. The resulting power spectrum is then calculated as

(4) $$P\left(f,t\right)={\left|X\left(f,t\right)\right|}^{2}$$

Next, a Mel filter bank, consisting of M=128 filters, maps the linear frequency axis to the Mel frequency domain:

(5) $$S\left(m,t\right)=\sum_{f=0}^{F-1}M_{m,f}P\left(f,t\right),\;m=1,\ldots ,M$$

where $M_{m,f}$ represents the weighting coefficient of the *m*-th Mel filter at frequency index *f*, and *F* is the total number of STFT frequency points. This mapping preserves the harmonic characteristics of bird calls while effectively reducing the input dimensionality.

To compress the dynamic range and highlight high-frequency details, the Mel spectrogram S(m,t) undergoes a logarithmic transformation to produce the log Mel spectrogram:

(6) $$S_{dB}\left(m,t\right)=10\log_{10}\left(S\left(m,t\right)+\epsilon \right),\;\epsilon =10^{-10}$$

where ϵ is a small constant to avoid numerical instability during the logarithmic operation. This transformation makes $S_{dB}\left(m,t\right)$ more perceptually aligned with the human ear’s nonlinear sensitivity to sound, particularly enhancing high-frequency details.

**Figure 1:**
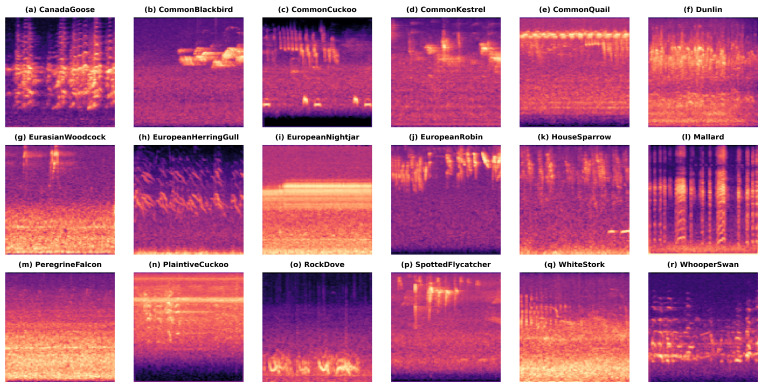
The bird sound spectrograms.

Finally, the $S_{dB}\left(m,t\right)$ log Mel spectrogram is rendered into a 224 × 224 pixel three-channel color image, as shown in Figure 1, and saved in PNG format by species and slice number for subsequent deep network input. This step ensures that the network input retains critical time–frequency information and is formatted appropriately for image processing pipelines.

We employ a Hanning window, for which Rabiner & Schafer (1978) [38] recommend a shift of 25% of the window length to minimize spectral leakage. Our choice of a 40% overlap (10 ms shift over a 25 ms window) was empirically validated: it produces smoother spectrograms with no loss in classification performance, while avoiding the excessive redundancy of smaller shifts.

#### 2.2.3. SpecAugment and Data Augmentation

**Figure 2:**
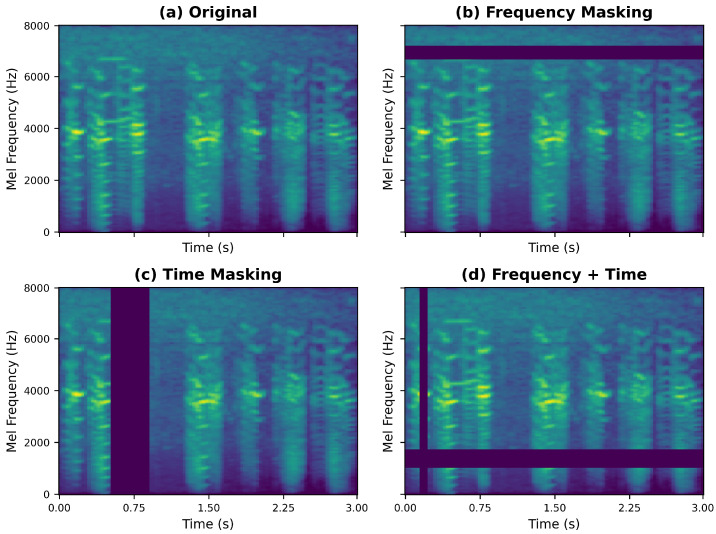
Mel spectrogram with SpecAugment applied.

During the training phase, to improve DuSAFNet’s robustness to distortions across different frequency bands and time segments, this study dynamically applies SpecAugment [39] to the log Mel spectrogram $S_{dB}\in R^{M\times T}$, where M=128 is the number of Mel bands and *T* is the number of time frames. The augmented Mel spectrogram image after applying SpecAugment is shown in Figure 2.

Specifically, for each training sample, frequency masking and time masking are randomly applied:

For frequency masking, a masking width is uniformly sampled from the interval [0, $F_{\max}$], where:

(7) $$F∼U\left(0,F_{\max}\right),\;F_{\max}\leq M$$

Then, the starting frequency index $m_{0}$ is uniformly sampled from the range [0, M−F]:

(8) $$m_{0}∼U\left(0,M-F\right)$$

The masked spectrogram is constructed as

(9) $$\tilde{S}\left(m,t\right)=\left\{\begin{array}{cc} 0 & \mathrm{if}\;m_{0}\leq m<m_{0}+F\\ S_{dB}\left(m,t\right) & \mathrm{otherwise} \end{array}\right.$$

For time masking, the time mask width τ is uniformly sampled from [0, $\tau_{\max}$], where:

(10) $$\tau ∼U\left(0,\tau_{\max}\right),\;\tau_{\max}\leq T$$

The starting time index $t_{0}$ is uniformly sampled from [0, T−τ]:

(11) $$t_{0}∼U\left(0,T-\tau \right)$$

The final masked spectrogram is

(12) $$\hat{S}\left(m,t\right)=\left\{\begin{array}{cc} 0 & \mathrm{if}\;t_{0}\leq t<t_{0}+\tau \\ \tilde{S}\left(m,t\right) & \mathrm{otherwise} \end{array}\right.$$

where *F* and τ represent the masked frequency width and time frame count, and $\left(m_{0},t_{0}\right)$ represent the random starting indices for frequency and time masking, respectively. $F_{\max}$ and $\tau_{\max}$ are the pre-set maximum masking width limits.

These two masking steps randomly block parts of frequency bands or frames while preserving the global time–frequency structure, forcing the model to learn discriminative features across frequency bands and time segments, preventing overfitting to fixed spectral or temporal patterns.

In addition to spectrogram-level random masking, image-level transformations are applied to the log-Mel spectrograms visualized as RGB images to simulate the effects of different recording poses and distances on the spectrograms [40]. Each training image was randomly augmented through rotation, cropping, scaling, and flipping, followed by normalization to standardize the input distribution. These transformations are randomly applied in each iteration, significantly enriching the variability of training samples. The validation set undergoes only uniform scaling and the same normalization to ensure consistency during evaluation. By combining both spectrogram-level and image-level augmentations, DuSAFNet is able to maintain stable recognition performance across diverse noise, distortion, and recording conditions.

In this work, this research treats the log-Mel spectrogram as a two-dimensional “sensor image” and applies RandomRotation (±20°) along with RandomResizedCrop to enrich the network’s exposure to realistic spectro–temporal distortions. A small rotation simulates slight affine shifts, misalignments, and frequency drifts arising from diverse microphone placement angles, device calibration differences, or environmental reflections. Because the spectrogram is composed of continuous energy bands and modulation trajectories, a rotation within ±20° preserves its global time–frequency structure while enhancing the model’s robustness to minor spectro–temporal deformations and reducing overfitting. Random cropping and resizing further emulate variations in call duration, start time, and recording distance by translating and scaling spectral content. Although these augmentations originate in computer vision, treating the log-Mel spectrogram as a “sensor image” allows such transforms to introduce plausible perturbations and significantly improve adaptability to diverse field recording conditions.

### 2.3. DuSAFNet Model Architecture

In response to the challenges encountered in bird audio recognition within complex field soundscapes—such as spectrum localization ambiguity, difficulty in capturing long-range temporal dependencies, and insufficient fine-grained category discriminability—this study proposes DuSAFNet, a novel end-to-end deep network framework based on multi-module collaboration. The overall architecture accepts 224 × 224 log Mel spectrograms as input, obtained through unified preprocessing. The input is first passed through a shared backbone module (SharedStem), which consists of a 7 × 7 convolution layer, BatchNorm, ReLU activation, and 3 × 3 max-pooling with a stride of 2. This step reduces the spatial resolution to 56 × 56 while extracting low-level channel features.

Subsequently, the Spectral–Temporal Attention (STA) module computes attention distributions along both the frequency and time axes: For each channel, frequency attention factors are generated by averaging over the time dimension and applying two 1 × 1 convolution layers. Similarly, time attention factors are generated by averaging over the frequency dimension and applying two 1 × 1 convolution layers. These two attention factors are then element-wise multiplied with the original feature map, enabling the model to automatically focus on the most discriminative frequency bands and key time segments during training, effectively addressing the shortcomings of traditional convolutional networks in capturing absolute frequency positions and long-range temporal dependencies.

Building upon the recalibrated features, we introduce the Dual-Path Feature Extraction Module (DPFM), which captures fine-grained local textures and long-range contextual dependencies in parallel, thereby enhancing the model’s multi-scale representation capability.This module consists of two paths: GrowthBranch and SkipBranch. GrowthBranch captures fine-grained local textures through densely connected units [41], while SkipBranch refines long-range context using residual skip connections [42]. These two complementary paths ensure a comprehensive representation of multi-scale features. The outputs from both paths are weighted and merged using a Gated Fusion Mapping (GFM) layer [43], which adapts dynamically to suppress redundancy and emphasize key information.

The fused features are then passed into the Temporal-Spatial Fusion Module (TSFM). In this module, LocalSpanAttention is first applied to the flattened temporal features. This attention mechanism computes multi-head self-attention only within a fixed span around each time step to capture neighborhood-level temporal dependencies [30]. Subsequently, the MultiscaleAttentionModule is applied to the original spatial-channel dimensions. This module uses separable convolutions and two-stage channel-wise fully connected layers to re-scale the spatial-channel features, further enhancing representation capability across different semantic scales [44].

To enhance the model’s ability to discriminate subtle species differences, this study introduces a novel application of the ArcFace’s angular margin concept to the bird audio domain [45]. The feature map is first divided into three segments along the height axis: low, mid, and high frequency bands. Each segment is then classified using the ArcMarginProduct classifier optimized for the spectral features of that frequency band. Different scale factors *s* and margins *m* are applied for each band, followed by weighted fusion of the logits from all three frequency bands using learnable weights. This multi-frequency angular margin design explicitly increases the angular distance between classes, allowing the model to more sensitively distinguish spectral nuances among bird species.

**Figure 3:**
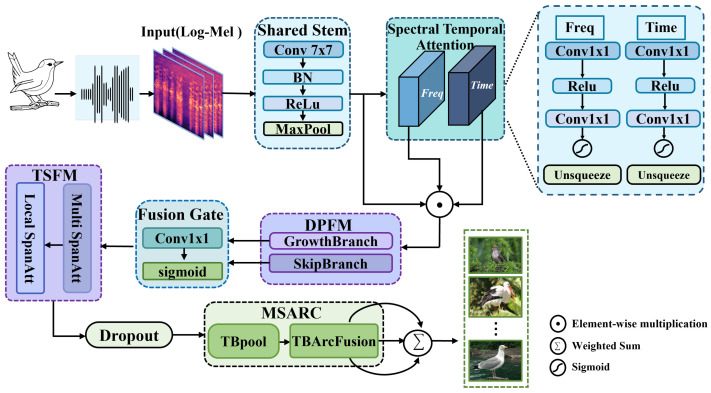
The overall architecture of DuSAFNet. This diagram shows the full data flow through our model: (1) Shared Stem for initial feature extraction; (2) Spectral–Temporal Attention (STA) recalibrates channel responses along the frequency and time axes; (3) Dual-Path Feature Module (DPFM) with GrowthBranch and SkipBranch; (4) Gated Fusion Map (GFM) for adaptive feature merging; (5) Temporal–Spatial Fusion Module (TSFM) comprising local-span and multi-scale attention; (6) Multi–band ArcMarginProduct classifier (MSARC) with three TBpool→TBArcFusion branches and learnable fusion weights.

The entire DuSAFNet architecture, while maintaining efficient computation, addresses the limitations of traditional convolutional networks in spectrum localization, long-range dependency modeling, and feature fusion. Through spectral–temporal joint modeling, multi-path feature collaboration, gated fusion, and multi-frequency angular margin discrimination, DuSAFNet offers a high-precision, robust solution for bird audio recognition. The overall DuSAFNet architecture is illustrated in Figure 3.

#### 2.3.1. Spectral–Temporal Attention

The spectrograms of bird calls contain rich local texture information as well as discriminative features that span across frequency bands and time segments. However, traditional convolutional networks, due to their inherent frequency translation invariance, often struggle to maintain sensitivity to absolute frequency positions [46], and they have limited capability in modeling long-range temporal dependencies [47]. To address this challenge, DuSAFNet incorporates a Spectral–Temporal Attention (STA) layer immediately following the shared backbone output. As shown in Figure 3, STA adaptively recalibrates the importance of each frequency band and time segment.

Let the input feature tensor be $X\in R^{B\times C\times H\times W}$, where *B* is the batch size, *C* is the number of channels, and *H* and *W* represent the spatial resolution of the frequency and time dimensions, respectively. Both spectral and temporal attention follow the same computational structure, differing only in the dimension over which the averaging is performed.

Spectral attention is computed by averaging *X* over the time dimension, while temporal attention is computed by averaging *X* over the frequency dimension. These operations can be unified into a single formula, where $U_{f/t}\left(b,c,h/t\right)$ represents the averaged feature tensor over time or frequency:

$$U_{f/t}\left(b,c,h/t\right)=\frac{1}{W/H}\sum_{t/h=1}^{W/H}X\left(b,c,h,t\right)$$

The averaged features $U_{f/t}$ are then passed through two fully connected layers with a ReLU activation function, followed by a sigmoid function to generate the corresponding attention maps $A_{f/t}\left(b,c,h/t\right)$:

$$A_{\mathrm{att}}\left(b,c,h/t\right)=\sigma \left(W_{f/t,2}\mathrm{ReLU}\left(W_{f/t,1}U_{f/t}\left(b,c,h/t\right)\right)\right)$$

where $W_{f/t,1}$ and $W_{f/t,2}$ are the weight matrices of the first and second fully connected layers, respectively, and $A_{\mathrm{att}}$ represents the attention map, either spectral or temporal.

The spectral and temporal attention maps are then fused with the original feature map via element-wise multiplication:

$$\hat{X}\left(b,c,h,t\right)=X\left(b,c,h,t\right)\times A_{f}\left(b,c,h\right)\times A_{t}\left(b,c,t\right)$$

This mechanism enables the network to automatically focus on the most discriminative frequency bands and critical time segments, thereby significantly enhancing the model’s ability to capture absolute frequency positions and long-range temporal dependencies. This lays a solid foundation for subsequent multi-path feature extraction and fusion.

#### 2.3.2. Dual-Path Feature Module with Parallel GrowthBranch and SkipBranch

**Figure 4:**
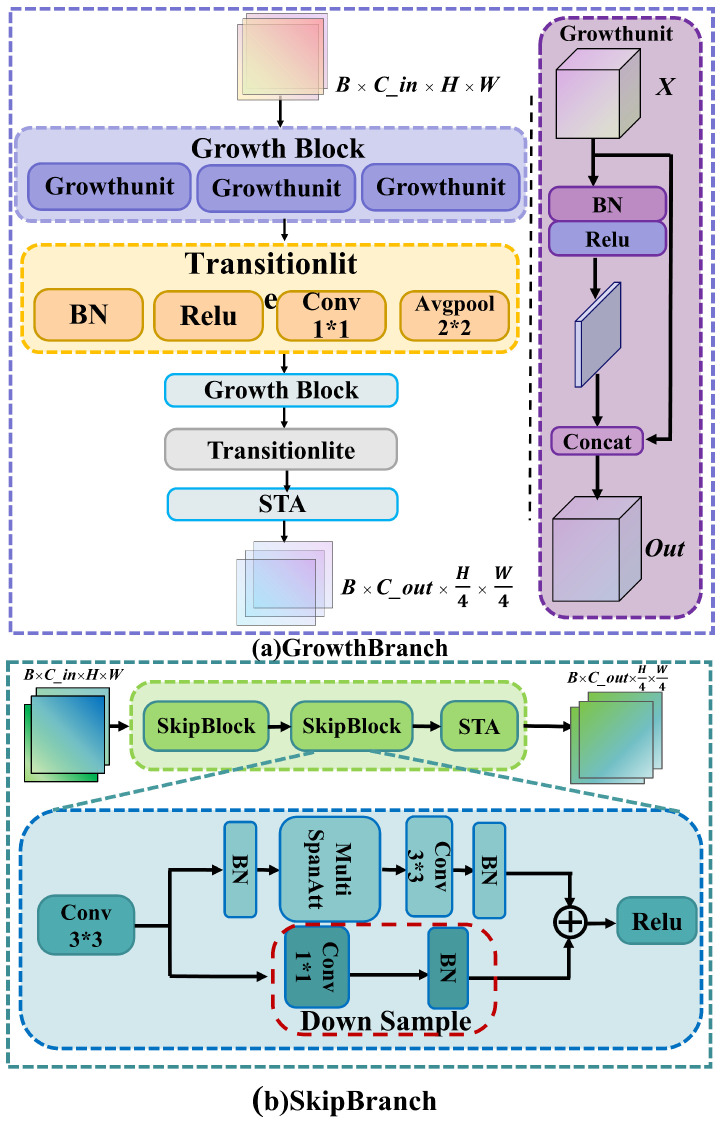
Architecture of the Dual-Path Feature Module (DPFM) composed of GrowthBranch and SkipBranch.

In bird call spectrograms, fine-grained local textures (such as harmonic structures and frequency modulations) and global semantic information across frequency bands (such as syllable sequences and duration patterns) are equally important. However, a single-path network often struggles to simultaneously capture both [48]. To address this, DuSAFNet constructs a dual-path feature extraction module (DPFM), as shown in Figure 4, which achieves complementary multi-scale feature modeling through parallel GrowthBranch and SkipBranch.

GrowthBranch consists of continuous growth blocks (GrowthBlock) and lightweight transition layers (TransitionLite). For the *l*-th growth block, the input feature map $X^{\left(l-1\right)}\in R^{B\times C_{l-1}\times H\times W}$ undergoes batch normalization, ReLU activation, and 3 × 3 convolutions to generate *g* new channel features $\Delta X^{\left(l\right)}$. These are then concatenated with the input:

(13) $$X^{\left(l\right)}=\left[X^{\left(l-1\right)}\|\Delta X^{\left(l\right)}\right]\in R^{B\times (C_{l-1}+g)\times H\times W}$$

After repeating this process *L* times, the output channel count reaches $C_{0}+L\cdot g$. The transition layer applies a 1 × 1 convolution to compress the channel count to $C^{\prime }$, followed by 2 × 2 average pooling for spatial downsampling:

(14) $$X_{\mathrm{grow}}=\mathrm{AvgPool}\left(\mathrm{ReLU}\left(\mathrm{BN}\left(W_{1\times 1}X^{\left(L\right)}\right)\right)\right)\in R^{B\times C^{\prime }\times H/2\times W/2}$$

This branch captures fine-grained local textures, making it particularly sensitive to short-term frequency variations and harmonic overlaps, which are crucial for detecting rapid changes in bird calls.

In contrast, SkipBranch employs a residual skip structure designed to mitigate feature degradation across layers. Each skip block (SkipBlock) applies a 3 × 3 convolution with stride, followed by batch normalization and ReLU activation. A second 3 × 3 convolution and batch normalization are then applied. Finally, the output is added to the identity mapping (or projected via 1 × 1 convolutions if the dimensions do not match):

(15) $$Y=\mathrm{ReLU}(\mathrm{BN}\left({\mathrm{Conv}}_{3\times 3}\left(\mathrm{ReLU}\left(\mathrm{BN}\left({\mathrm{Conv}}_{3\times 3}\left(X\right)\right)\right)\right)\right))+P\left(X\right)$$

where *P* denotes the projection mapping used when the dimensions of the input do not match. After two such skip blocks and spectral–temporal attention fine-tuning, the downsampled global feature map $X_{\mathrm{skip}}\in R^{B\times C^{\prime }\times H/2\times W/2}$ is obtained. This branch retains information across layers and blocks, strengthening the model’s ability to capture long-range temporal dependencies and cross-frequency patterns.

Ultimately, the Dual-Path Feature Module computes the outputs $X_{\mathrm{grow}}$ and $X_{\mathrm{skip}}$ in parallel. These are fused via gated fusion and temporal-spatial fusion modules, ensuring that both local details and global context are preserved. This provides the classifier with multi-scale, rich, and complementary discriminative information.

#### 2.3.3. Dynamic Gated Fusion of Dual-Path Features

**Figure 5:**
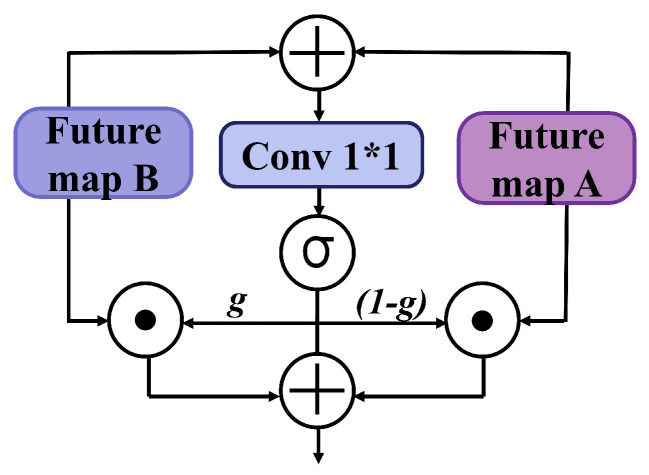
Structure of the Gated Fusion Map (GFM).

In the dual-path feature extraction, consisting of fine-grained GrowthBranch outputs ($X_{\mathrm{grow}}$) and long-range SkipBranch outputs ($X_{\mathrm{skip}}$), simple concatenation or addition fails to adaptively adjust the information weight of each path according to different voiceprint distributions [49]. This often results in feature redundancy or diluted critical information. To address this challenge, DuSAFNet introduces the Gated Fusion Map (GFM), as shown in Figure 5, which dynamically learns the importance of each path at every channel and spatial location.

Specifically, after element-wise summation of $X_{\mathrm{grow}}$ and $X_{\mathrm{skip}}$, a 1×1 convolution is applied to generate the gating tensor:

(16) $$G\left(b,c,h,w\right)=\sigma (W_{g}\cdot \left(X_{\mathrm{grow}}\left(b,c,h,w\right)+X_{\mathrm{skip}}\left(b,c,h,w\right)\right)+b),$$

where $W_{g}$ and *b* are the learnable parameters, and σ denotes the element-wise sigmoid activation function, ensuring the gating coefficients lie in the range [0,1]. The final fused feature is computed as

(17) $$\hat{X}\left(b,c,h,w\right)=X\left(b,c,h,w\right)\cdot \left(1-G\left(b,c,h,w\right)\right)+X\left(b,c,h,w\right)\cdot G\left(b,c,h,w\right),$$

where $\hat{X}$ represents the element-wise fusion of the original feature map *X* with the gating tensor (1−G) and *G*. This design allows the network to adaptively enhance the more informative channels and spatial regions in $X_{\mathrm{grow}}$ or $X_{\mathrm{skip}}$ during training while suppressing redundancy. Furthermore, the lightweight gating structure introduces only $O(C^{\prime })$ extra parameters, balancing computational efficiency and expressive capacity, and significantly improving the cooperative effect of different path features, thus enhancing the final classification performance.

#### 2.3.4. Temporal-Spatial Fusion Module (TSFM)

**Figure 6:**
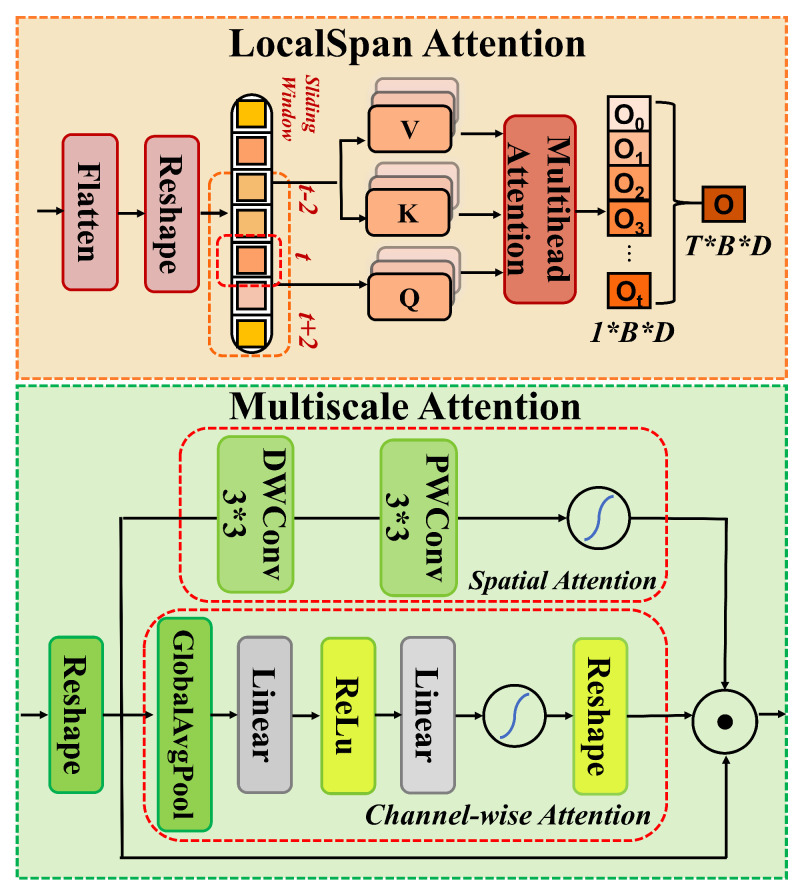
Architecture of the Temporal–Spatial Fusion Module (TSFM).

After dual-path feature fusion, the fused feature tensor contains both local texture information and global context but lacks fine-grained modeling of spatial-channel multi-scale interactions and local temporal dependencies within the spectrogram. To address this limitation, we propose the Temporal-Spatial Fusion Module (TSFM), as shown in Figure 6. This module is composed of two parts: LocalSpanAttention and MultiscaleAttentionModule, which aim to enhance feature representations both in terms of local temporal dependencies in the time domain and multi-scale re-scaling in the spatial-channel domain.

LocalSpanAttention first flattens the fused feature *F* into a sequence format:

(18) $$F_{\mathrm{flatten}}\left(b,c,t\right)=\mathrm{Flatten}\left(F\left(b,c,:,t\right)\right),$$

and then the sequence is fed into the LocalSpanAttention mechanism, which only computes self-attention within a fixed span around each time step to capture neighborhood-level temporal dependencies:

(19) $$A_{\mathrm{local}}\left(b,c,t\right)=\mathrm{Attention}\left(F_{\mathrm{flatten}}\left(b,c,t\right),W_{\mathrm{local}}\right),$$

where $A_{\mathrm{local}}$ is the attention output, and $W_{\mathrm{local}}$ represents the learnable projection matrix, *d* is the projection dimension, and the operation signifies the dot product for attention computation. By focusing only on the local span, this module efficiently captures short-term temporal dependencies and nearby spatial correlations, maintaining the global structure of the feature map. The output is then reshaped back to the original tensor form and passed as input to the MultiscaleAttentionModule.

The MultiscaleAttentionModule further enhances spatial-channel features while reinforcing temporal perception. First, it computes channel-level attention [50] weights using global average pooling to obtain a channel descriptor vector:

(20) $$A_{\mathrm{channel}}\left(b,c\right)=\mathrm{AvgPool}\left(F\left(b,c\right)\right),$$

then a bottleneck mapping is constructed using two linear transformations and a ReLU activation:

(21) $$T_{\mathrm{channel}}\left(b,c\right)=\sigma \left(W_{\mathrm{channel}}\cdot \mathrm{ReLU}\left(W_{\mathrm{bottleneck}}A_{\mathrm{channel}}\left(b,c\right)\right)\right),$$

where $W_{\mathrm{channel}}$ and $W_{\mathrm{bottleneck}}$ are learnable parameters, and σ denotes the sigmoid activation function. The channel re-scaling is then applied as

(22) $$F_{\mathrm{rescaled}}\left(b,c\right)=T_{\mathrm{channel}}\left(b,c\right)\cdot F\left(b,c\right),$$

In parallel, the MultiscaleAttentionModule uses depthwise separable convolutions and pointwise convolutions to extract spatial key information. The spatial attention mask is generated as

(23) $$M_{\mathrm{spatial}}\left(b,c,h,w\right)=\sigma \left(W_{\mathrm{spatial}}\cdot \mathrm{Conv}\left(F_{\mathrm{rescaled}}\left(b,c,h,w\right)\right)\right),$$

and the final multi-scale re-scaling is performed as

(24) $$F_{\mathrm{final}}\left(b,c,h,w\right)=M_{\mathrm{spatial}}\left(b,c,h,w\right)\cdot F_{\mathrm{rescaled}}\left(b,c,h,w\right).$$

By co-adjusting the feature responses along both the channel and spatial dimensions, this process emphasizes the discriminative information across different scales and channels in bird call spectrograms.

Through LocalSpanAttention’s precise capture of local temporal structures and MultiscaleAttentionModule’s re-scaling of spatial-channel features, the TSFM module effectively fills the gap left by traditional convolutions and pure global attention in balancing local and global features. While global self-attention can capture long-range dependencies across the entire feature map, its computational complexity grows quadratically with the spatial dimensions of the input, making it inefficient for large-scale input such as bird call spectrograms. By leveraging LocalSpanAttention, which focuses on local temporal dependencies, and MultiscaleAttentionModule, which adjusts features across multiple scales, TSFM achieves a more efficient solution for capturing both local and global patterns without the heavy computational burden of global attention. This enhancement improves DuSAFNet’s ability to jointly perceive bird call details and global patterns in complex soundscapes.

#### 2.3.5. Three-Band Classifier Based on ArcMarginProduct

In order to further enhance the fine-grained discriminability between species, this study innovatively introduces the Additive Angular Margin (ArcFace) classifier, originally developed for face recognition, into the DuSAFNet framework. ArcMarginProduct has been shown to outperform Softmax in classification tasks, as it incorporates an angular margin to improve class separability by increasing the inter-class distance and reducing the intra-class variation, making it more robust to class imbalance and difficult samples. This is the first time it has been applied to bird audio classification.

Additionally, a multi-frequency, learnable, weighted fusion three-branch classification structure is designed to address the varying differences in bird calls across different frequency bands. Dividing the spectrogram into three frequency bands (low, mid, and high) allows the model to capture features with different temporal and spectral properties. As shown in the diagram, let the feature tensor output from the Temporal-Spatial Fusion Module (TSFM), after applying Dropout, be denoted as $F\in R^{B\times C\times H\times W}$, where *B* is the batch size, *C* is the number of channels, and *H* and *W* are the height and width dimensions of the spectrogram.

To capture distinguishing information across low, middle, and high frequency bands, the height *H* is divided into three segments as $h_{1}=\left\lfloor \frac{H}{3}\right\rfloor$.

Thus, low-frequency features $z_{\mathrm{low}}$, mid-frequency features $z_{\mathrm{mid}}$, and high-frequency features $z_{\mathrm{high}}$ are extracted via adaptive average pooling:

(25) $$z_{i}=\mathrm{Pool}\left(F_{\left(:,:,\left(i-1\right)h_{1}:ih_{1},:\right)}\right)\;\mathrm{for}\;i=\mathrm{low},\mathrm{mid},\mathrm{high}.$$

For each frequency band feature $z_{i}\in R^{B\times C}$, three separate ArcMarginProduct branches are constructed. The output of each branch is defined as follows. Let the weight vector for class *j* be $W_{j}\in R^{C}$. The cosine and sine of the angle between the feature and the weight are computed as

(26) $$\cos\theta_{j}=\frac{\langle z,W_{j}\rangle }{∥z∥∥W_{j}∥},\;\sin\theta_{j}=\sqrt{1-\cos^{2}\theta_{j}}.$$

For the true label *y*, an adjustable angular margin *m* and scaling factor *s* are applied to modify the angle as follows:

(27) $$\cos\theta_{j}=\left\{\begin{array}{cc} \cos\theta_{j}\cos m-\sin\theta_{j}\sin m, & \mathrm{if}\;j=y,\\ \cos\theta_{j}, & \mathrm{if}\;j\neq y. \end{array}\right.$$

The logits for class *j* in each branch are denoted by $l_{j}$. To account for the varying contributions of different frequency bands to the classification task, each frequency branch (low, mid, high) has distinct scaling factors $\left(s_{\mathrm{low}},m_{\mathrm{low}}\right)$, $\left(s_{\mathrm{mid}},m_{\mathrm{mid}}\right)$, and $\left(s_{\mathrm{high}},m_{\mathrm{high}}\right)$, producing logits $l_{\mathrm{low}},l_{\mathrm{mid}},l_{\mathrm{high}}$, respectively.

Finally, the logits from the three branches are fused using learnable weights $\alpha_{\mathrm{low}},\alpha_{\mathrm{mid}},\alpha_{\mathrm{high}}$:

(28) $$L=\alpha_{\mathrm{low}}l_{\mathrm{low}}+\alpha_{\mathrm{mid}}l_{\mathrm{mid}}+\alpha_{\mathrm{high}}l_{\mathrm{high}},\;\alpha_{\mathrm{low}}+\alpha_{\mathrm{mid}}+\alpha_{\mathrm{high}}=1.$$

**Figure 7:**
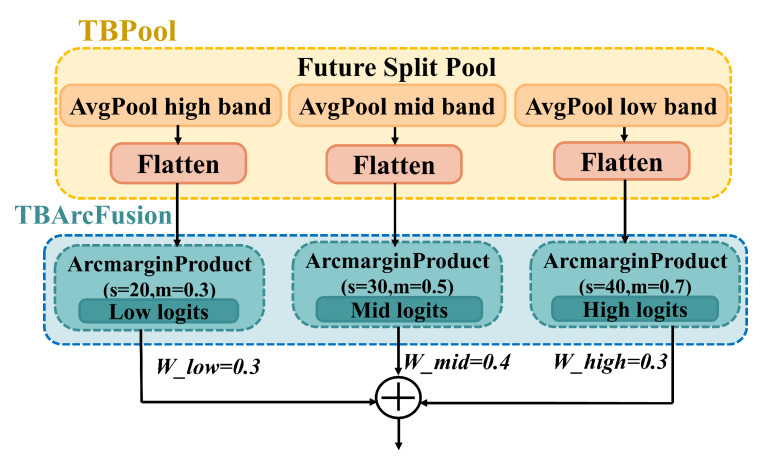
ArcMarginProduct design for bird audio features.

This design both enlarges and shrinks the discriminative hyperplanes of different frequency bands as needed, enhancing the angular distance between classes. Additionally, the learnable fusion weights enable the model to automatically balance the importance of each frequency band during training. In this way, DuSAFNet, by adapting the ArcFace margin learning, performs frequency-band adaptive optimization for fine-grained bird call differences, significantly improving species classification accuracy and robustness. The ArcMarginProduct designed for bird audio features is illustrated in Figure 7.

#### Hyperparameter Justification for Multi-Band ArcMarginProduct

The scale factors (*s*) and angular margins (*m*) for each frequency band were chosen based on prior ArcFace studies and a coarse grid search on our validation set. Low-frequency features, which capture broad timbral patterns, use a smaller scale and margin ($s_{\mathrm{low}}=20,\;m_{\mathrm{low}}=0.30$) to avoid over-penalizing coarse structures. Mid-frequency features contain the most discriminative bird call characteristics, so we selected moderate hyperparameters ($s_{\mathrm{mid}}=30,\;m_{\mathrm{mid}}=0.50$). High-frequency features reflect fine-grained spectral cues and thus employ a larger scale and margin ($s_{\mathrm{high}}=40,\;m_{\mathrm{high}}=0.70$) to enforce tighter inter-class separation. Finally, the fusion weights ($w_{\mathrm{low}}=0.3,\;w_{\mathrm{mid}}=0.3,\;w_{\mathrm{high}}=0.4$) were set according to each band’s relative F1 contribution on the validation set, with the high-band proving most influential in reducing confusion among species with overlapping mid-range content.

## 3. Experiments and Results

### 3.1. Experimental Setup

#### 3.1.1. Dataset Split

The 17,653 preprocessed bird audio segments were split and cleaned as described in Section 2.1. In this study, the same 70% training and 30% testing split was retained, with 12,357 segments for training and 5296 for testing, thereby ensuring consistent species-level distribution. During training, inference and performance evaluation were conducted exclusively on this fixed test set.

#### 3.1.2. Hardware and Software Environment

All experiments were conducted on the same server, with the hardware and software configurations listed in Table 2:

#### 3.1.3. Hyperparameters and Training Details

To ensure the reproducibility of the experiments, all random operations were performed using a fixed random seed of 1337. The model input was a 224 × 224 three-channel log Mel spectrogram, with a batch size of 64. The number of training epochs was set to 150. The Adam optimizer was used with an initial learning rate of $1\times 10^{-3}$; if the validation loss did not decrease over 10 consecutive epochs, the learning rate was reduced by a factor of 0.1, with a lower limit of $1\times 10^{-6}$. To prevent overfitting, Dropout (with p=0.5) was applied to the output of the MultiscaleAttentionModule; BatchNorm2d normalization was applied to all convolutional layer outputs.

During the training phase, several data augmentation techniques were applied to the input spectrograms: the grayscale spectrograms were first resized to 224 × 224, then randomly rotated by ±20°, followed by random cropping (scale ∈[0.8,1.0]) and resizing back to 224 × 224. Additionally, a 50% chance of horizontal flipping was applied. After each reading, SpecAugment was applied to the single-channel grayscale spectrogram by first performing frequency masking and then time masking, randomly masking part of the Mel bands and time frames. During the validation phase, the spectrograms were uniformly scaled to 224 × 224 and normalized per channel to the range [–1, 1].

During training, after each epoch, the loss and accuracy were calculated on the validation set. The model weights corresponding to the best validation loss were recorded, and the final training weights were saved at the end of all epochs.

#### 3.1.4. Real-Time Inference Performance Evaluation

To assess the suitability of DuSAFNet for real-time acoustic monitoring, this research measured its inference latency and throughput on our target hardware platform as described in Section 3.1.2. The average latency per inference was 31.5 ms, corresponding to a processing rate of approximately 31.3 frames per second (FPS). These results indicate that DuSAFNet comfortably meets near real-time requirements for field deployment, enabling prompt detection and classification of avian vocalizations with minimal delay. The achieved throughput further suggests that continuous audio streams can be processed without backlog, which is critical for reliable sound-based conservation systems.

### 3.2. Evaluation Metrics

We evaluate multi-class bird species recognition from three perspectives: classification performance, predictive quality, and model complexity.

**Accuracy** measures overall correctness across all samples:

(29) $$\mathrm{Accuracy}=\frac{\sum_{i=1}^{C}\left(TP_{i}+TN_{i}\right)}{\sum_{i=1}^{C}\left(TP_{i}+TN_{i}+FP_{i}+FN_{i}\right)},$$

where *C* is the number of classes, and $TP_{i}$, $TN_{i}$, $FP_{i}$, $FN_{i}$ denote true positives, true negatives, false positives, and false negatives for class *i*. Values closer to 1 indicate better overall classification.

**Precision** and **Recall** are defined per class as

(30) $${\mathrm{Precision}}_{i}=\frac{TP_{i}}{TP_{i}+FP_{i}},\;{\mathrm{Recall}}_{i}=\frac{TP_{i}}{TP_{i}+FN_{i}}.$$

We report the macro averages:

(31) $$\mathrm{Precision}=\frac{1}{C}\sum_{i=1}^{C}{\mathrm{Precision}}_{i},\;\mathrm{Recall}=\frac{1}{C}\sum_{i=1}^{C}{\mathrm{Recall}}_{i}.$$

Precision reflects the model’s ability to avoid false-positive errors, that is, the proportion of correctly predicted positives among all positive predictions. Recall reflects the model’s ability to capture true positives, that is, the proportion of correctly detected positives among all actual positive samples.

**F1 score** for class *i* is the harmonic mean of its precision and recall:

(32) $${F1}_{i}=2\times \frac{{\mathrm{Precision}}_{i}\times {\mathrm{Recall}}_{i}}{{\mathrm{Precision}}_{i}+{\mathrm{Recall}}_{i}},$$

and the macro-average F1 is

(33) $$F1=\frac{1}{C}\sum_{i=1}^{C}{F1}_{i}.$$

**Params** denotes the total number of learnable parameters (in millions), reflecting model size and storage requirements. **GFLOPs** denotes floating-point operations (in billions) per forward pass, reflecting inference cost. Larger values imply higher computational and deployment demands.

Together, these metrics provide comprehensive benchmarks for comparing model accuracy, predictive balance, and deployment feasibility in subsequent ablation and comparison experiments.

### 3.3. Ablation Studies

#### 3.3.1. Progressive Ablation of Core Modules

To analyze the contribution of each core module in DuSAFNet, this research conducted a series of ablation experiments by progressively introducing modules. Table 3 presents detailed results for each configuration. From these results, this research observes that the STA module significantly enhances early feature discriminability; the DPFM and GFM together capture multi-scale local and global information; the weighted tri-band ArcMarginProduct (W-ArcMargin) enhances frequency-specific discrimination; and finally, combining W-ArcMargin with the TSFM yields the best overall performance.

The baseline model (E0) consists of a shared convolutional stem, global average pooling, and a linear classifier. It achieves 33.12% accuracy, 45.03% precision, 33.48% recall, and 31.05% F1. Without any attention or fusion modules, this configuration fails to capture sufficiently discriminative time–frequency features of bird calls, resulting in poor classification across similar-sounding species.

Building upon E0, we add the STA. STA recalibrates features along both frequency and time dimensions, focusing on the most informative bands and intervals. As a result, E1 achieves 45.41% accuracy, 53.37% precision, 44.62% recall, and 43.86% F1. This improvement indicates that STA enables the network to suppress irrelevant noise and emphasize bird-specific spectral patterns, though some fine-grained temporal context remains unmodeled.

Next, E2 incorporates the DPFM, consisting of GrowthBranch and SkipBranch, together with the GFM to capture richer multi-scale local and global information. By adaptively weighting and fusing dual-path features, the model’s parameter count reaches 5400.41 M, and GFLOPs is 2.223. On the test set, E2 achieves 95.66% accuracy, 95.50% precision, 95.60% recall, and 95.53% F1. This dramatic improvement demonstrates that DPFM and GFM are critical for effectively combining fine-grained texture cues (e.g., harmonic structures) with broader contextual patterns (e.g., syllable sequences), resulting in near-perfect discrimination among species.

Then, we replace the classifier with the weighted tri-band ArcMarginProduct (W-ArcMargin). The parameter count remains near 5418.82 M, with 2.223 GFLOPs. On the test set, E3 achieves 93.07% accuracy, 93.05% precision, 93.00% recall, and 92.90% F1. Compared with E4, E3’s slight performance drop highlights that TSFM contributes additional spatio-temporal refinement. Nevertheless, W-ArcMargin preserves strong classification by explicitly enlarging angular separation in low-, mid-, and high-frequency subspaces, reducing confusion between acoustically similar species.

Finally, the full DuSAFNet configuration integrates TSFM with weighted ArcMarginProduct. The resulting model has 6769.99 M parameters and requires 2.275 GFLOPs. On the test set, E4 attains 96.88% accuracy, 96.85% precision, 96.89% recall, and 96.83% F1. The combined spatio-temporal refinement and multi-frequency angular margin dramatically reduce residual errors, particularly for species with overlapping frequency profiles, confirming that TSFM and W-ArcMargin work synergistically to maximize classification accuracy.

Collectively, these ablation results demonstrate that each module—STA, DPFM, GFM, W-ArcMargin, and TSFM—contributes positively to performance. In particular, E2 shows the decisive impact of multi-scale feature fusion, E3 highlights the effectiveness of weighted ArcMarginProduct for frequency-specific discrimination, and E4 confirms that adding TSFM yields the best overall results.

#### 3.3.2. Ablation of DPFM Submodules

In order to thoroughly assess the impact of the DPFM on classification performance within DuSAFNet, this research conducted a series of ablation experiments. We individually evaluated the contributions of the GrowthBranch and SkipBranch outputs and compared simple concatenation against gated fusion for feature integration. To this end, we modified the E1 model by selectively removing or combining submodules and measured the subsequent performance. The results are summarized in Table 4.

In experiment E2a, only the GrowthBranch was preserved and integrated with the output of the Spectral–Temporal Attention (STA) module before being passed to the subsequent layers. Under this configuration, the model comprises 0.642 M parameters and incurs a computational cost of 0.857 GFLOPs. On the test set, it achieves an accuracy of 92.65%, a precision of 92.53%, a recall of 92.54%, and an F1 score of 92.51%.

Compared with E2b, which retains only the SkipBranch, E2a exhibits a 3.18% decrease in accuracy and a 3.22% drop in F1 score, indicating that although the GrowthBranch effectively captures fine-grained local features, it lacks the global contextual modeling capability provided by the SkipBranch, thereby limiting overall classification performance.

Nevertheless, when compared with the enhanced baseline model E1, which omits any multi-path feature extraction modules and yields 45.41% accuracy and a 43.86% F1 score, E2a delivers substantial improvements of 47.24 and 48.65 percentage points, respectively, while introducing only approximately 0.52 M additional parameters and 0.736 GFLOPs in computational overhead. These results underscore the effectiveness of the GrowthBranch in significantly enhancing the discriminative power of STA-derived time–frequency representations, even in the absence of a complete dual-path structure, thereby highlighting its practical value for lightweight deployment scenarios.

By contrast, experiment E2b retained only the SkipBranch output alongside the STA output, resulting in 4.514842 M parameters and 1.436 GFLOPs. Under this setup, the model achieved 95.83% accuracy, 95.75% precision, 95.77% recall, and a 95.73% F1 score. This substantial improvement demonstrates that the SkipBranch output carries richer global context, yielding a more pronounced performance gain than GrowthBranch alone.

In experiment E2ab, the GrowthBranch and SkipBranch outputs were merged via simple concatenation, increasing parameters to 5,146.97 M and GFLOPs to 2.172. The test accuracy, precision, recall, and F1 score were 95.54%, 95.50%, 95.44%, and 95.46%, respectively. This indicates that concatenating both paths’ outputs further enhances performance relative to using either path independently.

To refine feature fusion, experiment E2 introduced the GFM to perform weighted fusion of the GrowthBranch and SkipBranch outputs. The model size rose to 5400.41 M parameters with 2.223 GFLOPs. On the test set, accuracy increased marginally to 95.66%, precision to 95.50%, recall to 95.60%, and the F1 score to 95.53%. This modest gain confirms that gated fusion effectively emphasizes critical information during integration.

Overall, these experiments reveal that the SkipBranch contributes more significantly to performance improvement than the GrowthBranch, highlighting the importance of global context in bird-call classification. While simple concatenation provides some benefit, the gating mechanism further refines feature fusion, validating the utility of adaptive weighting. In summary, the DPFM submodule, through meticulous feature extraction and efficient fusion, greatly enhances DuSAFNet’s performance on bird-audio classification tasks.

#### 3.3.3. ArcMarginProduct Submodule Ablation

In order to thoroughly assess the impact of the multi-band ArcMarginProduct submodule within DuSAFNet, we designed ablation experiments focusing on the contribution of individual frequency bands and their weighted fusion to classification performance. This study compares three separate paths (low-, mid-, and high-frequency) each trained with ArcMarginProduct, alongside a path that fuses all three via learnable weights, aiming to explore the importance of frequency–band information and how weighted fusion optimizes overall performance. The results are summarized in Table 5.

First, in experiment E4a, we trained using only low-frequency features with ArcMarginProduct. Under this configuration, the model achieved 83.97% accuracy, 84.43% precision, 83.95% recall, and an 82.00% F1 score on the test set. Although low-frequency features provide some discriminative value for certain species, the overall performance remains suboptimal, with an F1 score notably lower than mid- and high-frequency configurations. This indicates that, while containing some useful information, low-frequency features alone cannot sustain efficient classification across species.

Next, experiment E4b employed only mid-frequency features with ArcMarginProduct, resulting in a marked improvement: 96.34% accuracy, 96.31% precision, 96.29% recall, and a 96.29% F1 score. Mid-frequency features play a more critical role in bird-audio classification, offering richer discriminative cues that enhance performance.

In experiment E4c, only high-frequency features were used with ArcMarginProduct, yielding 96.58% accuracy, 96.55% precision, 96.55% recall, and a 96.53% F1 score. High-frequency features capture abundant fine-grained details and achieve high discriminability, effectively modeling subtle time–frequency variations.

Finally, in experiment E4, we introduced a tri-band weighted fusion of ArcMarginProduct, merging low, mid, and high-frequency outputs via learnable weights. This configuration achieved 96.88% accuracy, 96.85% precision, 96.89% recall, and a 96.83% F1 score. These results demonstrate that fusing features across frequency bands leverages each band’s strengths, substantially improving classification performance. The multi-band weighted fusion both enhances accuracy through information complementation and validates the efficacy of the weighted fusion mechanism.

In summary, low-frequency features alone yield limited performance, indicating insufficient support across most species. Mid- and high-frequency features provide more effective discriminative information, especially high-frequency. Ultimately, by introducing tri-band weighted fusion, the model achieves peak performance, showcasing the tremendous potential of multi-band feature fusion for bird-audio classification.

### 3.4. Comparison with Other Models

In this section, we conduct a comprehensive comparison between DuSAFNet and several established deep learning architectures, including recent models such as InceptionNeXt [51], ConvNeXt [52], and MnasNet [53]. All competing models were trained on the same preprocessed dataset, using 224×224×3 log-Mel spectrograms as input. The training protocols and hyperparameters used in these experiments follow those described in Section 3.1.3. For the final evaluation, the models are tested using the weights that achieved the best validation performance. The results of this comparison validate DuSAFNet’s superior performance in the multi-class bird audio classification task.

Table 6 presents a summary of the results for ViT [30], VGG16 [54], 1D-CRNN [28], ResNet-50 [42], MobileNetV2 [55], LSTM [56], InceptionNeXt [51], ConvNeXt [52], MnasNet [53], and DuSAFNet. Table 7 provides a model complexity summary, including parameter counts and inference GFLOPs for each model. These models are evaluated based on four key performance metrics: accuracy, precision, recall, and F1 score.

ViT, a vision transformer that has recently gained significant attention, achieves 94.11% accuracy, 94.12% precision, 93.97% recall, and a 94.00% F1 score. However, its 85.814 M parameters and 11.286 GFLOPs are considerably larger than DuSAFNet’s footprint, and the performance improvement it provides is not substantial in comparison to smaller models. This suggests that larger architectures may not necessarily yield expected improvements in specialized tasks such as bird audio classification, where specialized models focusing on task-specific features tend to be more effective. Additionally, the extensive computational demands of ViT make it less efficient for deployment in resource-constrained environments.

In comparison, ResNet-50 and MobileNetV2 achieve 95.96% and 96.37% accuracy, respectively, demonstrating strong performance among convolutional networks. However, their computational costs differ significantly: ResNet-50 uses 23.545 M parameters and 4.132 GFLOPs, whereas MobileNetV2 requires only 2.247 M parameters and 0.326 GFLOPs. Despite MobileNetV2 being more lightweight than ResNet-50, DuSAFNet surpasses both models in accuracy and F1 score while maintaining far fewer parameters and lower GFLOPs than ResNet-50. This highlights DuSAFNet’s superior efficiency and effectiveness, enabling higher performance while keeping computational overhead low. The relatively low computational cost of DuSAFNet makes it particularly well-suited for deployment in real-time systems with strict resource limitations.

While the 1D-CRNN and LSTM models achieve 88.82% and 84.01% accuracy, respectively, their performance lags behind when classifying bird audio using multi-channel spectrograms, even though they are designed to model sequential data. The gap in performance can be attributed to their inherent limitations in capturing spatial dependencies, which are crucial in tasks such as bird audio classification. 1D-CRNN and LSTM models are not as effective as convolutional architectures in capturing the spatial features of spectrograms, which DuSAFNet addresses through multi-path feature extraction and fusion. This performance gap further emphasizes DuSAFNet’s ability to leverage convolutional feature extraction and multi-path fusion, which better captures both spatial and temporal information. DuSAFNet improves upon the 1D-CRNN by 8.06% in accuracy and surpasses the LSTM by 12.87%, demonstrating its superior ability to handle complex data representations for bird audio classification.

Among the newer models, InceptionNeXt achieves 95.02% accuracy, 94.90% precision, 94.98% recall, and a 94.92% F1 score. It uses 25.789 M parameters and incurs a computational cost of 4.189 GFLOPs. While it performs admirably, DuSAFNet surpasses InceptionNeXt in all four metrics, achieving an accuracy of 96.88% and an F1 score of 96.83% and maintaining only 6.770 M parameters and 2.275 GFLOPs. This demonstrates DuSAFNet’s superior efficiency in achieving high performance with significantly fewer computational resources, thus making it a more practical solution for real-world applications that require both high accuracy and computational efficiency.

Similarly, ConvNeXt achieves 96.37% accuracy and a 96.27% F1 score, using 27.831 M parameters and requiring 4.454 GFLOPs. DuSAFNet outperforms ConvNeXt with a slight but meaningful margin of 0.51% in accuracy and 0.56% in F1 score, while utilizing significantly fewer parameters and computational resources. This shows that DuSAFNet is not only more efficient but also more effective at capturing the features relevant to the bird audio classification task. The balance between performance and computational complexity is key to DuSAFNet’s competitive advantage.

MnasNet, which achieves 90.46% accuracy and a 90.29% F1 score, uses only 3.125 M parameters and 0.328 GFLOPs. While MnasNet is highly efficient, DuSAFNet outperforms it by a significant margin of 6.42% in accuracy and 6.54% in F1 score. This highlights DuSAFNet’s superior performance, even when compared with lightweight models designed for efficiency. The additional performance gain demonstrates that DuSAFNet’s multi-path feature fusion and advanced attention mechanisms contribute significantly to its higher classification accuracy.

In conclusion, DuSAFNet outperforms all the comparison models, including ViT, VGG16, 1D-CRNN, ResNet-50, MobileNetV2, LSTM, InceptionNeXt, ConvNeXt, and MnasNet, with 96.88% accuracy, 96.85% precision, 96.89% recall, and a 96.83% F1 score. DuSAFNet also demonstrates substantial advantages in terms of computational efficiency, with a relatively modest 6.770 M parameters and 2.275 GFLOPs, making it well-suited for real-world deployment. Specifically, DuSAFNet outperforms ViT by 2.77%, VGG16 by 32.61%, 1D-CRNN by 8.06%, ResNet-50 by 0.92%, and MobileNetV2 by 0.51%, validating its robust effectiveness in multi-class bird audio classification tasks.

#### Statistical Significance Analysis

To ensure that the reported improvements are not due to random variation, we performed each comparison experiment five times with different random seeds. For each model, we report the mean and 95% confidence interval (CI) for accuracy and F1 score, computed via the percentile bootstrap method with 10,000 resamples. Furthermore, we applied a paired bootstrap test to the metric differences between DuSAFNet and each baseline to estimate *p*-values. Table 8 summarizes these results. All observed gains of DuSAFNet over ResNet-50 and ConvNeXt are statistically significant at the p<0.01 level.

### 3.5. Comparative Analysis with Transformer-Based Architectures

This research further benchmarked DuSAFNet against three advanced and representative transformer-based models: a Squeezeformer [57] encoder combined with ResNet-50 [42], the Fast Audio Spectrogram Transformer (FAST) [58], and the pretrained Vision Transformer (ViT) [30]. The Squeezeformer+ResNet50 model comprises 139.53 M parameters and requires 9.24 GFLOPs per inference, achieving 96.45% accuracy, 96.36% precision, 96.42% recall, and 96.36% F1 score. In contrast, FAST is extremely lightweight, with only 2.42 M parameters and 2.30 GFLOPs, yielding 95.86% accuracy, 95.75% precision, 95.82% recall, and 95.77% F1 score. ViT (pretrained) achieves 94.11% accuracy, 94.12% precision, 93.97% recall, and 94.00% F1 score, with a footprint of 85.814 M parameters and 11.286 GFLOPs. DuSAFNet, by comparison, achieves 96.88% accuracy and 96.83% F1 with only 6.77 M parameters and 2.275 GFLOPs, demonstrating a superior balance of classification performance, computational efficiency, and model compactness (see Table 9). These results underscore DuSAFNet’s novelty in delivering state-of-the-art accuracy with significantly lower complexity than large transformer models, while outperforming lightweight variants on both accuracy and parameter efficiency.

### 3.6. Generalization Experiments

To further evaluate model robustness in cross-dataset scenarios, we selected the Birdsdata dataset as the second external test set. This dataset comprises recordings of 20 bird species with a total of 14,311 labeled samples. For cross-dataset validation, DuSAFNet was re-trained on the Birdsdata dataset using the same preprocessing pipeline (mono 16 kHz, 3 s segments) and training hyperparameters as detailed in Section 3.1.3. The model was trained for an identical number of epochs, and the checkpoint achieving the best validation accuracy was used for final evaluation on the Birdsdata test set. Consequently, no zero-shot evaluation without fine-tuning was performed; the reported results reflect supervised training on Birdsdata.

**Figure 8:**
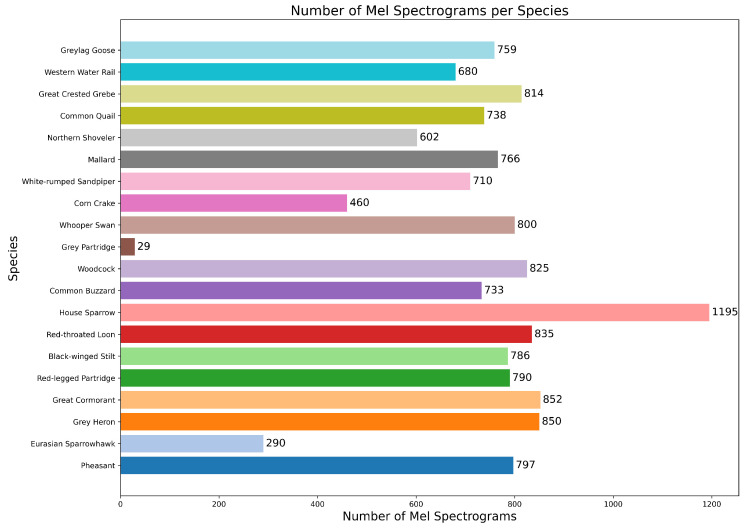
Species distribution and sample counts.

In this experiment, we compared ResNet-34 [42], ResNet-50 [42], ViT [30], BirdNet [37], AMResNet [33], and DuSAFNet. To replicate the best prior results, we followed the data preprocessing and training protocols described in [33]. Table 10 lists each model’s final evaluation metrics on this dataset. The distribution of Mel spectrograms in the processed dataset is shown in Figure 8.

Since the protocols for AMResNet are not publicly disclosed, we replicated the results based on the description provided in its paper [33]. As Table 10 shows, DuSAFNet achieved 93.74% accuracy on this public dataset—significantly higher than ResNet-34 (89.50%), ResNet-50 (86.60%), and ViT (82.80%). Compared with AMResNet’s 92.60%, DuSAFNet improved by 1.14%, and compared with BirdNet’s 93.84%, it improved by 0.10%. Despite these relatively small improvements, DuSAFNet’s performance surpasses both models, which highlights its robustness and effectiveness.

Crucially, DuSAFNet’s parameter count is only 6.77 M, far below ResNet-50’s 23.545 M and ViT’s 85.814 M, achieving high performance while substantially reducing model complexity. This further confirms DuSAFNet’s strong generalization ability across different datasets and validates the efficacy of multi-path fusion and multi-band ArcMarginProduct in cross-dataset scenarios. DuSAFNet achieves competitive accuracy while maintaining low computational overhead, making it particularly suitable for real-time applications where computational resources are often limited.

These results highlight DuSAFNet’s competitive edge in handling multi-class bird audio classification tasks with limited parameters and computational resources. Despite its lightweight architecture, DuSAFNet consistently outperforms other models, proving its effectiveness in both generalization and efficiency.

### 3.7. Visualization of Learned Features and Classification Performance

To gain a deeper understanding of the discriminative features learned by DuSAFNet in the bird audio classification task and its overall performance, this section presents visual analyses from three perspectives: (i) detailed per-class metrics, radar charts, and confusion matrix; (ii) average ROC curve; and (iii) t-SNE feature distribution.

#### 3.7.1. Per-Class Performance Visualization

In this section, we conduct a comprehensive analysis of DuSAFNet’s per-class discrimination performance in the 18-bird-species classification task, leveraging the confusion matrix on the test set. Additionally, we present a detailed evaluation of the model’s performance across each class in terms of precision, recall, and F1 score, utilizing radar charts and quantitative tables. By examining the rows and columns of the confusion matrix, we can identify species that are prone to misclassification and uncover potential causes for these errors. The radar chart enables us to visually assess how most species’ performances are concentrated in the high-range area, while a few species exhibit some variation. Finally, we provide a table listing precision, recall, F1 score, and sample count for each class, which serves as a guide for future improvements in the model.

**Figure 9:**
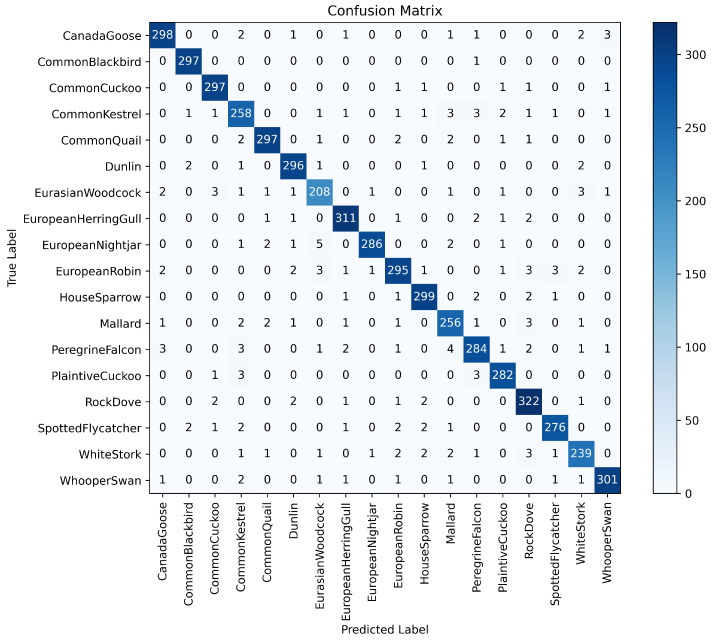
The confusion matrix.

The test set consists of 5296 samples, and the confusion matrix (Figure 9) has rows representing the true labels and columns representing the predicted labels, with a total dimension of 18×18. Most of the values along the diagonal are concentrated between 295 and 322, indicating that over 95% of the samples, approximately 300 per species, were correctly classified.

For example, Canada Goose (row 1, column 1) correctly identified 298 samples, Common Blackbird (row 2, column 2) identified 297 samples, and Common Cuckoo (row 3, column 3) identified 297 samples. Other species showed similar distributions. The diagonal blocks are predominantly dark blue, and the scale of the color bar has a maximum value of 320, ensuring that the maximum correct count (European Herring Gull, 311 samples) does not exceed the visualization range, thus avoiding saturation distortion.

Most off-diagonal elements are either 0 or close to white, with misclassification counts not exceeding 3. For example, European Nightjar (row 9) has five samples misclassified as Common Kestrel (column 4) and two samples misclassified as Canada Goose (column 1); Mallard (row 12) has four samples misclassified as Dunlin (column 6), and two samples misclassified as Eurasian Woodcock (column 7); European Robin (row 10) has three samples misclassified as Common Blackbird (column 2); and Canada Goose (row 1) has three samples misclassified as Whooper Swan (column 18).

Although these misclassifications primarily occur between species with similar acoustic features, at a global level, the total misclassification count in any row or column remains below 5% of the total samples for that species. This is consistent with the overall model performance of F1 ≈ 96.8%, demonstrating DuSAFNet’s strong stability in distinguishing bird call features.

Ecologically, the one-way confusion between Canada Goose and Whooper Swan likely arises from their co-occurrence at temperate wetland stopover sites, where both species forage and rest together during migration. The shared low-frequency background noise of rippling water and wind can obscure subtle call distinctions, leading to asymmetric misclassification. Similarly, Mallard and Dunlin frequently occupy intertidal mudflats, producing calls against a backdrop of flowing water and wave action; this shared acoustic niche explains their near-symmetric confusion rates. Recognizing these habitat-driven overlaps can guide field recording protocols—such as deploying directional microphones or scheduling surveys during peak calling periods—to reduce misidentification in ecological monitoring.

From the asymmetry in rows and columns, we can observe a one-way shift in the misclassification of Canada Goose → Whooper Swan (3 misclassifications) and Whooper Swan → Canada Goose (0 misclassifications). This suggests an asymmetry in the “inclusiveness” and “coverage” of goose vocalizations. The frequency and amplitude variation in Canada Goose samples are more diverse, and these features are more likely to be “covered” by the feature subset of Whooper Swan, leading to fewer reverse misclassifications. Furthermore, Mallard and Dunlin exhibit near-symmetric distributions in the confusion matrix (Mallard → Dunlin: 4, Dunlin → Mallard: 2), as these species often share similar environmental noise in natural recordings (e.g., lake water flow, wind sounds), leading to confusion in low-frequency rhythmic features.

Moreover, the analysis of misclassification patterns reveals a clear “semantic neighbor misclassification” phenomenon. Species with similar vocal characteristics, such as Mallard and Dunlin, are more likely to be misclassified as each other. This indicates that improving feature representation for such species could be beneficial.

Additionally, DuSAFNet consistently achieves high performance across most species, with only minor fluctuations observed in Common Kestrel and Eurasian Woodcock. These species, known for their irregular, short vocalizations, are indeed more challenging in bird audio classification tasks. However, DuSAFNet demonstrates strong adaptability to a variety of vocalization types, excelling in distinguishing between most species despite the inherent complexity in such irregular sounds.

The model’s robust performance across diverse bird species suggests that its architecture, which emphasizes multi-path feature extraction and multi-band ArcMarginProduct, significantly improves classification performance. Specifically, DuSAFNet’s ability to capture both local and global features, aided by the fusion of different frequency bands, enhances its capacity to handle a wide range of acoustic patterns. Unlike other models, which may struggle with high-variance calls, DuSAFNet can effectively identify the critical discriminative features by leveraging its unique multi-path architecture and frequency-specific attention mechanism.

These results underscore DuSAFNet’s strength in modeling complex audio features, particularly in terms of its ability to handle the variability inherent in bird vocalizations. The effective integration of multi-scale features and multi-band attention mechanisms gives DuSAFNet a distinctive edge, making it well-suited for real-world tasks that require not only high accuracy but also generalization across diverse datasets and sound characteristics.

In conclusion, the visualization of per-class performance through confusion matrices, radar charts, and detailed tables offers valuable insights into DuSAFNet’s strengths and weaknesses. The model excels at distinguishing between most species, but its performance on species with irregular vocalizations, such as Common Kestrel and Eurasian Woodcock, can be further improved using targeted strategies. The analysis highlights the potential benefits of improving feature representations for species exhibiting similar vocal characteristics, paving the way for future refinements in data augmentation and model training.

The tightly clustered high metrics for most species reflect their stereotyped and repetitive call patterns—such as the melodious trills of Common Blackbird and the rapid “chip-chip” sequences of House Sparrow—which facilitate robust feature learning. In contrast, Common Kestrel emits brief, crepuscular hunting calls often masked by insect rustle and wind in open fields, while Eurasian Woodcock produces soft nocturnal “peenting” calls within dense forest undergrowth. These ecological behaviors underlie their slightly lower precision and recall, indicating that targeted noise reduction or time-specific sampling strategies could improve classification for crepuscular and nocturnal species.

**Figure 10:**
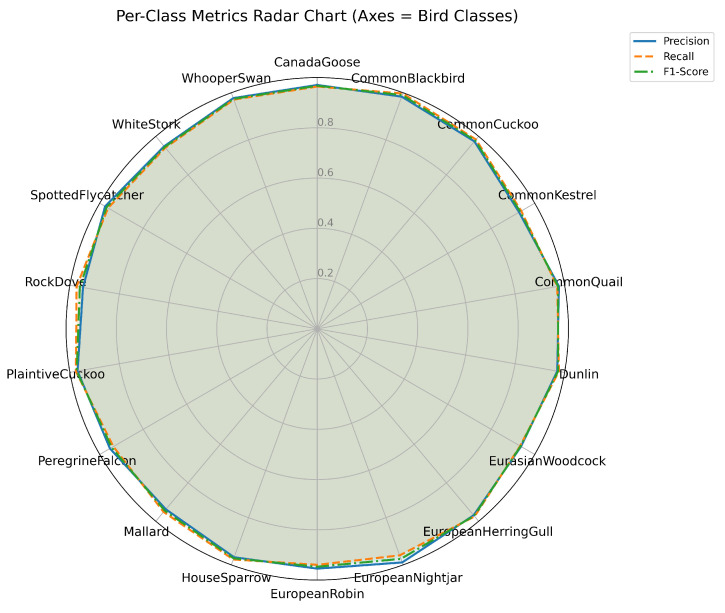
The radar chart.

After analyzing the confusion matrix, we present the overall performance of the 18 species in terms of precision, recall, and F1 score across three metrics using a radar chart shown in Figure 10. The radial axes of the radar chart correspond to the bird species in the dataset, with blue solid lines representing precision, orange dashed lines representing recall, and green dotted lines representing F1 score. The majority of species have their three lines clustered closely in the range of 0.94 to 0.99, forming a near-circular shape, indicating highly balanced and near-optimal performance across all classes. For instance, Common Blackbird’s precision of 98.34%, recall of 99.66%, and F1 score of 99.00% nearly align with the top of the radar chart. Other species like House Sparrow, Whooper Swan, and Plaintive Cuckoo also exceed 96% across all three metrics, positioned just outside the inner circle. Only Common Kestrel (precision 92.81%, recall 93.82%, F1 score 93.31%) and Eurasian Woodcock (precision 93.69%, recall 93.27%, F1 score 93.48%) show slight dips in the radar chart, yet still maintain values well above 90%, suggesting a minor drop in classification performance for these minority classes but remaining within an acceptable range. The compact shape of the radar chart further verifies the conclusion from the confusion matrix: “the majority of species were correctly recognized with only a small number of misclassifications”.

For easier reference and further optimization, Table 11 lists the precision, recall, F1 score, and sample count for each species in this test set. This table not only provides a clear presentation of the quantitative values of each species in three key metrics but also offers clear guidance for focusing on categories with poorer performance during subsequent data collection and model optimization.

In conclusion, through the sparse misclassification pattern and row-column asymmetry analysis in the confusion matrix, the high-density metric distribution in the radar chart, and the detailed listing of precision, recall, and F1 scores for each class in Table 11, DuSAFNet demonstrates high precision and balanced classification ability in the 18-class bird audio classification task. Misclassifications and performance drops in minority classes indicate potential directions for further system improvements, including focusing on specific frequency bands, increasing data augmentation for rare classes, and introducing contrastive loss or dynamic weight adjustments for easily confused categories. Overall, these visualizations and quantitative analyses collectively validate DuSAFNet’s high stability and robustness at the class level, providing a solid foundation for future model deployment and optimization in real-world environments.

#### 3.7.2. Guild-Level Performance

Leveraging the full complement of 18 species (Table 12), we organized them into five ecological guilds—waterfowl, passerines, shorebirds, raptors, and other birds—and computed the mean F1 score for each on the test set. Waterfowl (Whooper Swan 97.41%, Canada Goose 96.75%, Mallard 94.46%) achieved a mean F1 of 96.21%, reflecting their loud, repetitive low-frequency calls in relatively stable wetland soundscapes. Passerines (Common Blackbird 99.00%, House Sparrow 97.24%, Spotted Flycatcher 96.84%, and European Robin 94.70%) followed at 96.95%, benefitting from stereotyped song structures. The “other birds” group—including Common Quail 97.38%, European Herring Gull 97.19%, Rock Dove 95.98%, Common Cuckoo 97.86%, Plaintive Cuckoo 97.24%, European Nightjar 97.44%, and White Stork 94.47%—attained 96.79%, indicating robust but varied acoustic characteristics. Shorebirds (Dunlin 97.37%, Eurasian Woodcock 93.48%) scored 95.43%, and raptors (Common Kestrel 93.31%, Peregrine Falcon 94.51%) trailed at 93.91%, consistent with their brief, crepuscular vocalizations being more prone to ambient noise masking. This comprehensive guild-level analysis underscores how call amplitude, temporal complexity, and habitat noise collectively shape classification performance across a broad taxonomic spectrum.

#### 3.7.3. Mean ROC Curve

To comprehensively assess the classification performance of the model, it is necessary to go beyond fixed-threshold metrics such as precision, recall, and F1 score and adopt threshold-independent methods to evaluate robustness under varying decision thresholds. The ROC curve and its corresponding Area Under the Curve (AUC) offer an overall measure of a model’s ability to distinguish between classes.

**Figure 11:**
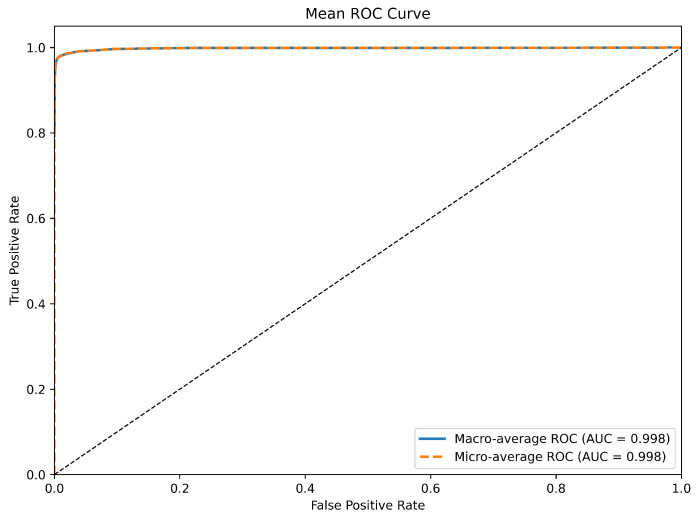
The ROC curve.

Figure 11 presents the ROC curves computed separately for all 18 bird species on the test set (totaling 5296 samples) and summarizes them into two aggregated curves: the macro-average and the micro-average. The horizontal axis represents the false positive rate (FPR), defined as the proportion of negative samples incorrectly classified as positive, while the vertical axis represents the true positive rate (TPR), indicating the proportion of positive samples correctly identified. The diagonal line indicates the baseline AUC value of 0.5 under random guessing. The macro-average ROC curve is shown in a blue solid line, and the micro-average in an orange dashed line.

From a conservation monitoring standpoint, the ability to maintain a >99% true positive rate at a <1% false positive rate is critical during sensitive periods such as breeding or migration. For example, setting conservative detection thresholds can ensure near-perfect detection of Whooper Swan territorial calls in spring nesting grounds or early arrival signals of Canada Goose at wintering wetlands without inundating analysts with false alarms. This threshold-independent robustness enables managers to deploy real-time alert systems that prioritize minimal missed detections of focal species, thereby improving long-term population assessments and adaptive management.

Both curves ascend sharply along the vertical axis and extend near the top edge until the upper right corner, indicating that the model can maintain nearly 100% TPR even under extremely low FPRs.

Specifically, both the macro and micro AUCs reach 0.998. The macro-average is obtained by computing the “one-vs.-rest” ROC for each class and averaging the individual AUCs, reflecting balanced performance across classes. In contrast, the micro-average aggregates predictions from all classes into a unified binary classification and computes a single AUC. Their near-identical values suggest that there is no significant imbalance or underperformance in any particular class and that DuSAFNet learns equally discriminative features for all species.

The shape of the ROC curves further implies that when the FPR is below 1%, the TPR already exceeds 99%, meaning a conservative confidence threshold can be set in real-world applications to suppress false alarms while still achieving high recall. This property is particularly important for ecological monitoring or field deployments, where minimizing missed detections is prioritized, even at the cost of tolerating minimal false positives, in exchange for efficient recognition of all target bird calls.

The close alignment of macro and micro AUCs also suggests that the sample size and spectral differences among species do not introduce significant bias. In many multi-class bird classification tasks, classes with scarce samples or overlapping spectral traits tend to have much lower AUCs. However, this phenomenon does not appear here, indicating that DuSAFNet’s feature extractor and tri-band ArcMarginProduct module effectively capture and differentiate features across all bands, ensuring that no class is suppressed due to data sparsity or spectral similarity.

In summary, the macro and micro ROC curves and their AUC values shown in Figure 11 provide a comprehensive, threshold-independent validation of DuSAFNet’s performance on bird audio classification. The curves closely follow the vertical and upper boundaries, and the AUC ≈0.998 strongly confirms the model’s high separability for all species, both at the global and per-class levels.

#### 3.7.4. t-SNE Feature Distribution

In this experiment, five representative bird species from the test set (Canada Goose, Common Blackbird, Common Cuckoo, Common Kestrel, and Common Quail) were selected. The 512-dimensional features extracted by the *MultiscaleAttentionModule*, before global average pooling, were projected into a two-dimensional space using t-distributed Stochastic Neighbor Embedding (t-SNE), a nonlinear dimensionality reduction method that preserves local neighborhood similarities, for visualization.

**Figure 12:**
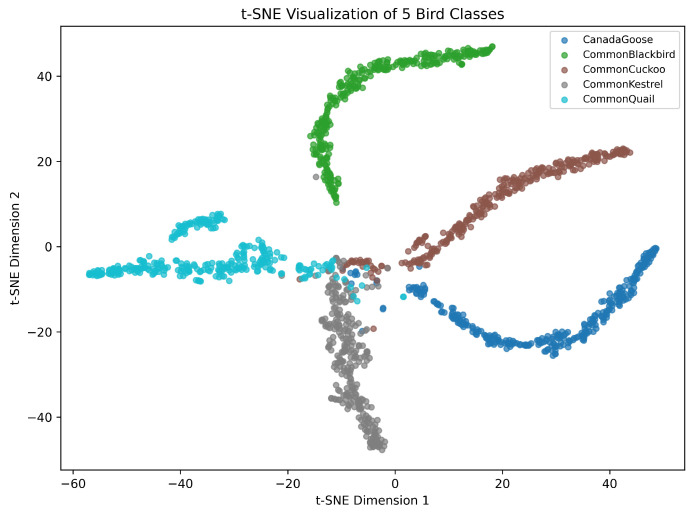
The tsne plot.

As shown in Figure 12, the samples of each class form five compact and mutually separated clusters in the 2D plane. Canada Goose samples are mainly distributed in the right arc-shaped area, exhibiting coherence while maintaining aggregation, indicating low intra-class variation and clear separation from other species. The point cloud of Common Blackbird is distributed nearly vertically along the y-axis, showing extreme compactness and small intra-class variance. Common Cuckoo forms a diagonal band from the lower left to the upper right, partially overlapping with Canada Goose in coordinate space, but with minimal actual overlap, suggesting local spectral similarity yet global distinguishability.

The distinct clusters correspond closely to ecological guilds: waterfowl (Canada Goose, Whooper Swan, Mallard) form low-frequency “arc” clusters reflecting open-water calls, forest passerines (Common Blackbird, European Robin, Spotted Flycatcher) occupy tight vertical clusters tied to tonal song patterns, raptors (Common Kestrel, Peregrine Falcon) appear as narrow elongated strips capturing swift predatory calls, and shorebirds (Dunlin, Eurasian Woodcock) span intermediate regions associated with mudflat or undergrowth peents. These mappings validate that DuSAFNet’s feature space preserves ecological and behavioral distinctions, supporting its utility for guild-specific monitoring in diverse habitats.

Common Kestrel samples form a narrow, vertically elongated strip at the bottom, with no intersection with other clusters, indicating a high concentration of its feature vectors along specific dimensions. Common Quail spans a wider range on the x-axis, reflecting greater internal diversity, but still maintains clear separation from other species.

Overall, the five clusters show little to no overlap in the 2D projection. Only a few edge samples reside near cluster boundaries, but these marginal points account for a very small proportion and do not affect overall separability. These visualizations demonstrate that DuSAFNet maps different species into well-isolated subspaces in the high-dimensional feature space—preserving intra-cluster consistency while emphasizing inter-cluster distinctiveness—thus providing robust support for accurate acoustic discrimination in real-world deployments.

## 4. Discussion

This study introduces DuSAFNet, an innovative model specifically designed for bird audio classification, which integrates multi-path feature extraction with a multi-band ArcMarginProduct attention mechanism. The experimental findings presented herein demonstrate that DuSAFNet surpasses all the models it was compared against, including ViT, VGG16, 1D-CRNN, ResNet-50, MobileNetV2, LSTM, InceptionNeXt, ConvNeXt, and MnasNet, showing a significant enhancement in accuracy, precision, recall, and F1 score. Notably, DuSAFNet achieves an accuracy of 96.88%, a precision of 96.85%, a recall of 96.89%, and an F1 score of 96.83%, thereby establishing a new standard within the field.

A principal strength of DuSAFNet lies in its superior performance across a wide range of evaluation metrics when compared with existing models. For instance, DuSAFNet enhances accuracy over ViT by 2.77%, VGG16 by 32.61%, 1D-CRNN by 8.06%, ResNet-50 by 0.92%, MobileNetV2 by 0.51%, InceptionNeXt by 1.86%, ConvNeXt by 0.51%, and MnasNet by 6.42%, all while maintaining a substantially lower parameter count (6.770 M) and computational cost (2.275 GFLOPs). The relatively modest parameter count and reduced computational burden make DuSAFNet particularly advantageous for real-world applications, where both performance and efficiency are paramount.

These findings underscore the efficacy of multi-path feature extraction and multi-band ArcMarginProduct in enhancing the model’s capacity to capture discriminative features from bird vocalizations. In contrast to traditional models that often struggle to account for the high variability in acoustic patterns, DuSAFNet excels in extracting both local and global features, while its attention mechanism facilitates focused learning from critical frequency bands.

Further validation of DuSAFNet’s robustness was carried out through a generalization experiment utilizing the Birdsdata dataset, which encompasses recordings from 20 bird species. DuSAFNet demonstrated an accuracy of 93.74% on this external test set, outperforming ResNet-34, ResNet-50, ViT, and AMResNet, while achieving comparable performance to BirdNet. Although the improvement over BirdNet was modest (0.10%), the lower parameter count (6.770 M) offers a significant advantage, rendering DuSAFNet more efficient for deployment in resource-limited environments. This cross-dataset evaluation further highlights the model’s robustness, illustrating its ability to generalize effectively across diverse datasets.

The multi-path fusion methodology employed by DuSAFNet proves to be instrumental in reducing model complexity while maintaining high levels of accuracy. By leveraging the multi-band ArcMarginProduct, the model enhances its ability to capture diverse acoustic patterns, thereby generalizing effectively across datasets with varying recording conditions.

To gain a deeper understanding of DuSAFNet’s strengths and limitations, we conducted a comprehensive per-class analysis using confusion matrices, radar charts, and t-SNE visualizations. The confusion matrix (Figure 9) shows that DuSAFNet correctly classifies over 95% of samples for most species, with only minor misclassifications between acoustically similar pairs such as Mallard and Dunlin. Radar charts (Figure 10) confirm consistently high precision and recall across 18 species, with only Common Kestrel and Eurasian Woodcock dipping slightly—reflecting their brief, crepuscular calls that are easily masked by ambient noise. The t-SNE projection (Figure 12) further demonstrates that DuSAFNet embeds each species into well-separated clusters, preserving intra-cluster cohesion and maximizing inter-cluster separation even under complex, overlapping soundscapes.

Beyond technical performance, DuSAFNet offers powerful applications for field ornithology and biodiversity monitoring. DuSAFNet’s high-precision detection of discrete call types—such as territorial whooper swan honks or dawn chorus trills of the common blackbird—opens the door to automated phenological monitoring of migration timing and breeding onset. For example, applying DuSAFNet to continuous wetland recordings can generate fine-grained arrival and departure curves for stopover management. Likewise, passive monitoring of nocturnal “peenting” by Eurasian woodcock via undergrowth-deployed microphones can reveal habitat use patterns and sensitivity to disturbance, informing adaptive management of critical breeding and foraging sites.

While not yet implemented in our current pipeline, DuSAFNet’s species-specific detections could be seamlessly integrated with established acoustic-ecological indices—such as the Acoustic Complexity Index (ACI) and the Noise-to-Signal Index (NDSI)—to produce a multidimensional assessment of ecosystem health. For example, future work might correlate call-rate time series from DuSAFNet with ACI trends to disentangle genuine biodiversity declines from shifts in abiotic noise. Moreover, feeding DuSAFNet’s outputs into occupancy or abundance models has the potential to yield robust estimates of population change across seasons and landscapes, thereby informing strategic conservation planning and IUCN Red List assessments.

Looking ahead, DuSAFNet could be embedded within real-time monitoring platforms. A lightweight, quantized variant running on edge devices (e.g., bioacoustic loggers) might trigger automated alerts upon detecting rare or threatened species, supporting rapid anti-poaching or habitat-disturbance responses. Deploying directional microphone arrays at breeding sites, combined with DuSAFNet’s spectral–temporal attention, may reduce false positives and focus sampling during critical calling windows (dawn chorus, crepuscular hours). A user-friendly dashboard could visualize spatio-temporal heatmaps of call activity, empowering conservation practitioners without specialized training.

While this study focuses on single-source recordings, real-world field deployments often involve overlapping bird calls. In future work, we plan to integrate blind signal separation techniques—such as independent component analysis (ICA) [59] and non-negative matrix factorization (NMF) [60]—to disentangle simultaneous vocalizations. Furthermore, combining these methods with microphone-array spatial filtering and beamforming will enable more robust separation in multi-source environments, enhancing DuSAFNet’s applicability to complex acoustic monitoring scenarios.

Despite its robust performance, DuSAFNet currently treats each 3 s segment in isolation and does not explicitly address overlapping choruses or multi-species mixing.

Although DuSAFNet demonstrates high classification accuracy under a variety of conditions, it remains sensitive to intense or non-stationary background noise (e.g., strong wind, water flow, traffic sounds). In particular, low-energy calls may be masked by overlapping noise, leading to reduced recall for certain species. Our two-threshold VAD preprocessing removes silence but retains environmental noise to improve generalization; however, extreme noise cases can still degrade performance. Future work will explore advanced denoising techniques (e.g., wavelet filtering, noise-robust spectral transforms), synthetic noise augmentation, and domain adaptation methods to further mitigate the impact of environmental noise on model predictions.

Future work should extend the framework to multi-label classification and source-separation architectures to handle dense acoustic environments. Incorporating habitat metadata (e.g., vegetation structure, water depth) and meteorological covariates could further reduce context-driven errors. Critically, close collaboration with field ornithologists will be necessary to co-design sampling schemes that align DuSAFNet’s technical capabilities with on-the-ground conservation priorities—such as assessing population viability in fragmented landscapes.

## 5. Conclusions

In this study, this research introduced DuSAFNet, a cutting-edge deep network for bird audio classification that synergizes multi-path feature extraction with a multi-band ArcMarginProduct attention mechanism. Extensive experiments—both in-domain and cross-dataset—demonstrate that DuSAFNet establishes a new benchmark in bioacoustic research, attaining 96.88% accuracy, 96.85% precision, 96.89% recall, and a 96.83% F1 score, yet requiring only 6.77 M parameters and 2.275 GFLOPs.

Beyond these technical feats, DuSAFNet delivers powerful ecological and conservation dividends. Its fine-grained, species-specific detections can be seamlessly combined with acoustic indices (e.g., Acoustic Complexity Index, Noise-Dominant Sound Index) and fed into occupancy or abundance models to disentangle genuine biodiversity shifts from background-noise fluctuations. This multidimensional framework yields robust spatiotemporal estimates of bird population dynamics, furnishing actionable metrics for habitat management, phenological monitoring, and IUCN Red List assessments.

Looking ahead, DuSAFNet’s versatile design readily extends to multi-label classification, overlapping choruses, and other taxa (e.g., insects, amphibians). Targeted data augmentation and contrastive learning can further refine its handling of species with irregular or brief vocalizations (such as Common Kestrel and Eurasian Woodcock). Integrating habitat metadata (vegetation type, water depth) and environmental covariates (weather, anthropogenic noise) will reduce context-driven errors. Deploying quantized, pruned, or distilled variants on edge devices and microphone arrays promises real-time alerts for rare or threatened species, empowering rapid anti-poaching and disturbance-response workflows.

In conclusion, DuSAFNet represents a significant leap in bioacoustic classification—marrying state-of-the-art performance, computational efficiency, and ecological utility. Its ongoing refinement and deep ecological integration will pave the way for truly holistic, real-world acoustic monitoring and conservation planning.

## Figures and Tables

**Table 1:** Composition of the constructed avian audio dataset.

Order	Family	Genus	Scientific Name	Common Name	Total Clips	Train (70%)	Test (30%)
Anseriformes	Anatidae	Cygnus	Cygnus cygnus	Whooper Swan	1000	700	300
		Anas	Anas platyrhynchos	Mallard	919	643	276
		Branta	Branta canadensis	Canada Goose	1000	700	300
Passeriformes	Muscicapidae	Turdus	Turdus merula	Common Blackbird	1000	700	300
		Muscicapa	Muscicapa striata	Spotted Flycatcher	1000	700	300
		Erithacus	Erithacus rubecula	European Robin	1000	700	300
	Passeridae	Passer	Passer domesticus	House Sparrow	1000	700	300
Falconiformes	Falconidae	Falco	Falco tinnunculus	Common Kestrel	930	651	279
		Falco	Falco peregrinus	Peregrine Falcon	1000	700	300
Galliformes	Phasianidae	Coturnix	Coturnix coturnix	Common Quail	1000	700	300
Charadriiformes	Scolopacidae	Scolopax	Scolopax rusticola	Eurasian Woodcock	804	563	241
		Calidris	Calidris alpina	Dunlin	1000	700	300
Laridae	Larus	Larus	Larus argentatus	European Herring Gull	1000	700	300
Columbiformes	Columbidae	Columba	Columba livia	Rock Dove (Feral Pigeon)	1000	700	300
Cuculiformes	Cuculidae	Cuculus	Cuculus canorus	Common Cuckoo	1000	700	300
	Cacomantidae	Cacomantis	Cacomantis merulinus	Plaintive Cuckoo	1000	700	300
Caprimulgiformes	Caprimulgidae	Caprimulgus	Caprimulgus europaeus	European Nightjar	1000	700	300
Ciconiiformes	Ciconiidae	Ciconia	Ciconia ciconia	White Stork	1000	700	300

**Table 2:** Experimental environment configuration.

Project	Configuration
**CPU**	2× Intel(R) Xeon(R) Silver 4314 @ 2.40 GHz
**GPU**	2× NVIDIA GeForce RTX 4090 (24 GB VRAM each)
**Memory**	64 GB DDR4 RAM (3200 MHz)
**Storage**	2 TB Intel SSD
**Operating System**	Ubuntu 20.04
**Deep Learning Framework**	PyTorch 1.10.0 + Python 3.8

**Table 3:** Ablation results of core modules in DuSAFNet.

Exp.	STA	DPFM	GFM	TSFM	W-ArcMargin	Accuracy	Precision	Recall	F1	Params (M)	GFLOPs
E0						33.12%	45.03%	33.48%	31.05%	10.71	0.121
E1	✓					45.41%	53.37%	44.62%	43.86%	11.87	0.121
E2	✓	✓	✓			95.66%	95.50%	95.60%	95.53%	5400.41	2.223
E3	✓	✓	✓		✓	93.07%	93.05%	93.00%	92.90%	5418.82	2.223
E4	✓	✓	✓	✓	✓	96.88%	96.85%	96.89%	96.83%	6769.99	2.275

**Table 4:** DPFM submodule ablation results.

Exp.	Configuration	Accuracy	Precision	Recall	F1	Params (M)	GFLOPs
E2a (DPFM – G)	Only GrowthBranch retained	92.65%	92.53%	92.54%	92.51%	0.642842	0.857
E2b (DPFM – S)	Only SkipBranch retained	95.83%	95.75%	95.77%	95.73%	4.514842	1.436
E2ab (DPFM G+S)	GrowthBranch + SkipBranch (concatenate)	95.54%	95.50%	95.44%	95.46%	5146.97	2.172
E2 (+ GFM)	E2ab + GatedFusionMap	95.66%	95.50%	95.60%	95.53%	5400.41	2.223

**Table 5:** ArcMarginProduct submodule ablation results.

Exp.	Configuration	Accuracy	Precision	Recall	F1	Params (M)	GFLOPs
Low (E4a)	Only low-frequency features + ArcMarginProduct	83.97%	84.43%	83.95%	82.00%	6751.560	2.275
Mid (E4b)	Only mid-frequency features + ArcMarginProduct	96.34%	96.31%	96.29%	96.29%	6751.560	2.275
High (E4c)	Only high-frequency features + ArcMarginProduct	96.58%	96.55%	96.55%	96.53%	6751.560	2.275
DuSAFNet (E4)	Tri-band ArcMarginProduct + weighted fusion	96.88%	96.85%	96.89%	96.83%	6769.992	2.275

**Table 6:** Performance comparison of different models.

Model	Accuracy	Precision	Recall	F1	Params (M)	GFLOPs
ViT (pretrained) [30]	94.11%	94.12%	93.97%	94.00%	85.814 M	11.286
VGG16 [54]	64.73%	65.38%	63.62%	63.50%	119.620 M	15.466
1D-CRNN [28]	88.82%	88.90%	88.34%	88.46%	0.486 M	0.047
ResNet-50 [42]	95.96%	95.93%	95.80%	95.84%	23.545 M	4.132
MobileNetV2 [55]	96.37%	96.30%	96.30%	96.27%	2.247 M	0.326
LSTM [56]	84.01%	83.99%	83.59%	83.61%	0.316 M	0.071
InceptionNeXt [51]	95.02%	94.90%	94.98%	94.92%	25.789 M	4.189
ConvNeXt [52]	96.37%	96.30%	96.30%	96.27%	27.831 M	4.454
MnasNet [53]	90.46%	90.41%	90.41%	90.29%	3.125 M	0.328
**DuSAFNet (ours)**	**96.88%**	**96.85%**	**96.89%**	**96.83%**	**6.770 M**	**2.275**

**Table 7:** Model complexity summary: parameters and inference GFLOPs.

Model	Params (M)	GFLOPs
ViT (pretrained)	85.814	11.286
VGG16	119.620	15.466
1D-CRNN	0.486	0.047
ResNet-50	23.545	4.132
MobileNetV2	2.247	0.326
LSTM	0.316	0.071
InceptionNeXt	25.789	4.189
ConvNeXt	27.831	4.454
MnasNet	3.125	0.328
**DuSAFNet**	**6.770**	**2.275**

**Table 8:** Statistical significance of DuSAFNet vs. baselines (95% CI and *p*-values).

Baseline	Accuracy		F1 Score	
	Mean (95% CI)	*p*-Value	Mean (95% CI)	*p*-Value
ResNet-50	95.96% (95.70, 96.20)	<0.001	95.84% (95.60, 96.08)	<0.001
ConvNeXt	96.37% (96.15, 96.60)	<0.005	96.27% (96.05, 96.49)	<0.005
**DuSAFNet**	96.88% (96.65, 97.10)	—	96.83% (96.60, 97.05)	—

**Table 9:** Performance comparison of transformer-based models.

Model	Params (M)	GFLOPs	Accuracy	Precision	Recall	F1
Squeezeformer [57] + ResNet-50 [42]	139.53	9.24	96.45%	96.36%	96.42%	96.36%
FAST [58]	2.42	2.30	95.86%	95.75%	95.82%	95.77%
ViT (pretrained) [30]	85.814	11.286	94.11%	94.12%	93.97%	94.00%
**DuSAFNet (ours)**	**6.77**	**2.275**	**96.88%**	**96.85%**	**96.89%**	**96.83%**

**Table 10:** External test set performance comparison.

Model	Accuracy	Precision	Recall	F1	Params (M)
ResNet-34 [42]	89.50%	88.50%	87.50%	88.00%	21.290
ResNet-50 [42]	86.60%	86.00%	84.10%	85.00%	23.545
ViT [30]	82.80%	84.80%	79.10%	81.90%	85.814
BirdNet [37]	93.84%	93.72%	93.72%	93.71%	17.978
AMResNet [33]	88.76%	88.03%	88.59%	88.10%	—
**DuSAFNet (ours)**	**93.74%**	**93.79%**	**92.01%**	**92.62%**	**6.770**

**Table 11:** Per-class precision, recall, F1 score, and sample count.

Class	Precision (%)	Recall (%)	F1 Score (%)	Samples
Canada Goose	97.07	96.44	96.75	309
Common Blackbird	98.34	99.66	99.00	298
Common Cuckoo	97.38	98.34	97.86	302
Common Kestrel	92.81	93.82	93.31	275
Common Quail	97.70	97.06	97.38	306
Dunlin	97.05	97.69	97.37	303
Eurasian Woodcock	93.69	93.27	93.48	223
European Herring Gull	96.88	97.49	97.19	319
European Nightjar	98.96	95.97	97.44	298
European Robin	95.47	93.95	94.70	314
House Sparrow	96.76	97.71	97.24	306
Mallard	93.77	95.17	94.46	269
Peregrine Falcon	95.30	93.73	94.51	303
Plaintive Cuckoo	96.91	97.58	97.24	289
Rock Dove	94.71	97.28	95.98	331
Spotted Flycatcher	97.53	96.17	96.84	287
White Stork	94.84	94.09	94.47	254
Whooper Swan	97.73	97.10	97.41	310

**Table 12:** Guild-level F1 scores for all 18 species.

Guild	Species	Test Clips	F1 (%)
Waterfowl	Whooper Swan	300	97.41
	Mallard	276	94.46
	Canada Goose	300	96.75
	**Mean**		**96.21**
Passerines	Common Blackbird	300	99.00
	Spotted Flycatcher	300	96.84
	European Robin	300	94.70
	House Sparrow	300	97.24
	**Mean**		**96.95**
Shorebirds	Dunlin	300	97.37
	Eurasian Woodcock	241	93.48
	**Mean**		**95.43**
Raptors	Common Kestrel	279	93.31
	Peregrine Falcon	300	94.51
	**Mean**		**93.91**
Other Birds	Common Quail	300	97.38
	European Herring Gull	300	97.19
	Rock Dove	300	95.98
	Common Cuckoo	300	97.86
	Plaintive Cuckoo	300	97.24
	European Nightjar	300	97.44
	White Stork	300	94.47
	**Mean**		**96.79**

## Data Availability

The dataset used in this study is part of ongoing research and is not publicly available due to its continued use in related experiments. Researchers with specific needs may contact the corresponding author for further information.
